# Accuracy of Deep Learning in Diagnosing Chronic Obstructive Pulmonary Disease: Systematic Review and Meta-Analysis

**DOI:** 10.2196/83459

**Published:** 2026-01-14

**Authors:** Hui Yang, Yijiu Wu, Tong Wu, Jingyan Ji, Sitao Lei, Weibin Xu

**Affiliations:** 1 School of Management, Guizhou University, Huaxi District, Guiyang, Guizhou, 550025, China, 86 18286022086; 2 School of Forensic Medicine, Guizhou Medical University, Guiyang, Guizhou, China; 3 School of Anesthesiology, Guizhou Medical University, Guiyang, Guizhou, China

**Keywords:** chronic obstructive pulmonary disease, COPD, deep learning, diagnosis, Global Initiative for Chronic Obstructive Lung Disease, GOLD grading, meta-analysis

## Abstract

**Background:**

Chronic obstructive pulmonary disease (COPD) is a common chronic lung disease. Deep learning (DL), a data-driven machine learning approach, has gained attention in clinical practice, particularly for diagnosing COPD and grading its severity. However, systematic evidence of its diagnostic and grading accuracy remains limited, posing challenges for developing intelligent diagnostic tools.

**Objective:**

This study aimed to systematically estimate the accuracy of DL models for diagnosing and grading COPD, providing up-to-date evidence for the design and clinical implementation of intelligent detection tools.

**Methods:**

The Cochrane Library, Embase, Web of Science, and PubMed were systematically searched for studies on DL for diagnosing COPD and grading its severity published up to November 1, 2025. Risk of bias was assessed using the Quality Assessment of Diagnostic Accuracy Studies-2 tool. Subgroup analyses by the validation set generation method and imaging data source were conducted, and meta-analyses were performed on the validation sets. For binary outcomes, diagnostic 2×2 tables were synthesized using a bivariate mixed effects model; for multiclass outcomes, accuracy estimates were pooled using random-effects models.

**Results:**

In total, 56 studies comprising 886,753 participants were included. Inputs were computed tomography (CT) imaging (n=30), breath sounds or audio (n=12), conventional chest X-ray (n=2), X-ray film (n=2), and other modalities (n=10), including pulmonary function indices or curves or physiological waveforms, electrocardiograms, volumetric capnography maps, radiogenetic data, and clinical scores. For binary classification of COPD, DL models yielded a pooled sensitivity of 0.87 (95% CI 0.85‐0.90), specificity of 0.88 (95% CI 0.84‐0.92), diagnostic odds ratio (DOR) of 52 (95% CI 30‐88), and the area under the summary receiver operating characteristic curve (AUC) of 0.93. For CT-based DL models, pooled sensitivity was 0.86 (95% CI 0.84‐0.89), specificity was 0.87 (95% CI 0.82‐0.90), DOR was 42 (95% CI 26‐68), and AUC was 0.92. For respiratory sound–based models, sensitivity was 0.91 (95% CI 0.84‐0.95), specificity was 0.96 (95% CI 0.91‐0.98), DOR was 237 (95% CI 78‐723), and AUC was 0.98. In multiclass classification, the DL models showed limited accuracy in discriminating Global Initiative for Chronic Obstructive Lung Disease (GOLD) stages: GOLD stage 0 (84.2%, 95% CI 60.5%‐98.2%), stage 1 (61.7%, 95% CI 40.7%‐80.8%), stage 2 (67.9%, 95% CI 37.6%‐91.7%), stage 3 (70.8%, 95% CI 16.3%‐100%), and stage 4 (70.8%, 95% CI 16.3%‐100%).

**Conclusions:**

This study is the first systematic synthesis of DL applications for COPD detection and GOLD staging. DL models based on CT images and breath sounds show high accuracy for binary COPD detection, whereas multiclass GOLD grading remains concerning. These findings support the development and updating of artificial intelligence−assisted COPD screening tools; however, substantial heterogeneity and limited external validation warrant cautious interpretation. Future reproducible multicenter studies with standardized reporting are needed.

## Introduction

Chronic obstructive pulmonary disease (COPD) is a prevalent chronic respiratory illness characterized by persistent airflow limitation. It is irreversible and progressively worsens over time, severely affecting patients’ quality of life and life expectancy [1]. According to the latest World Health Organization report, COPD is the fourth leading cause of death worldwide, responsible for over 3 million deaths each year, leading to a disproportionate burden in low- and middle-income countries [2]. China accounts for about one-quarter of the global burden of COPD, with an estimated 99.9 million people affected and a prevalence of 13.7% among adults aged ≥40 years [3]. Acute exacerbations are pivotal events in COPD, causing hospital admission and increasing the risk of mortality. The 5-year mortality rate after exacerbation is about 50% after hospitalization [4]. A real-world multicenter prospective cohort study in Japan has reported a 5-year survival rate of 85.4% among COPD patients, whereas those with very severe airflow limitation have a reduced 5-year survival rate of 66.1% [5]. Consequently, COPD not only represents a significant public health issue worldwide but has also become one of the main causes of disability and death.

In clinical practice, the gold standard for diagnosing COPD is pulmonary function testing (PFT), which primarily quantifies expiratory airflow limitation. Based on the Global Initiative for Chronic Obstructive Lung Disease (GOLD) guidelines, COPD is defined as the ratio of forced expiratory volume in 1 second to forced vital capacity <0.70 (or the lower limit of normal for individuals of the same age, sex, and height), measured prior to and following bronchodilator use [6]. However, it is challenging to implement PFT. It requires specialized spirometry equipment and trained personnel, and participants must repeatedly perform forceful exhalation maneuvers. Older adults or severely ill patients often produce false-negative results due to insufficient effort. In addition, the procedure may induce coughing, dizziness, or other discomforts and poses a risk of cross-infection under pandemic conditions or in poorly controlled environments. These factors limit the application of PFT in community and primary care settings [7]. Thus, relying solely on conventional PFT is insufficient for screening COPD. Developing simpler, non-invasive, and more scalable auxiliary diagnostic methods for early detection of COPD is, therefore, imperative.

In recent years, deep learning (DL) has attracted significant attention in clinical practice. DL is a complex neural network framework. Common DL models include convolutional neural networks, residual networks, densely connected networks, inception networks, and vision transformer models [8]. These models excel at feature extraction and classification, allowing the automatic learning of high-level semantic information from large datasets, thereby markedly improving the precision and efficiency of image processing and signal analysis [9]. Although PFT is recognized as the gold standard for the auxiliary diagnosis of COPD, researchers often employ chest imaging (including computed tomography [CT] scans and X-rays) or respiratory sounds to develop DL-based alternative or complementary tools for improving diagnostic efficiency and convenience. However, these traditional methods heavily rely on researchers’ prior knowledge, and variations in diagnostic criteria and annotation practices across different teams result in significant heterogeneity, affecting the reproducibility and generalizability of diagnostic outcomes [10,11]. In this context, some studies have used DL for the automatic diagnosis of COPD, such as DL-based chest X-ray (CXR), for the classification of COPD [10] and DL-based cough sound signal analysis [11]. Nevertheless, systematic evidence of the actual performance and comparative advantages of different DL frameworks in the diagnosis of COPD is lacking.

Therefore, we conducted a systematic review and meta-analysis of diagnostic test accuracy studies on DL models for COPD. Our first objective was to describe the diagnostic performance of these models for identifying COPD across different data sources (such as CT images and respiratory sounds) in both internal and external validation sets. Our second objective was to assess the performance of DL models in classifying the severity of COPD, particularly GOLD stages. We hypothesized that DL models would show good accuracy for the diagnosis of COPD, whereas their performance for staging COPD would be more variable and less stable.

## Methods

### Study Registration

This systematic review and diagnostic test accuracy meta-analysis was conducted and reported in accordance with the PRISMA (Preferred Reporting Items for Systematic Reviews and Meta-Analyses) 2020 statement and the PRISMA-DTA (Preferred Reporting Items for a Systematic Review and Meta-analysis of Diagnostic Test Accuracy Studies) extension, and the search methods were reported following PRISMA-S (Preferred Reporting Items for Systematic reviews and Meta-Analyses literature search extension) [12,13]. The PRISMA-S checklist is provided in Checklist 1. The protocol was prospectively registered in PROSPERO (International Prospective Register of Systematic Reviews; CRD420251114195 [14]).

### Eligibility Criteria

The inclusion criteria were as follows: (1) original research that developed a DL model for diagnosing COPD or classifying COPD severity; (2) studies reported at least one of the following outcome measures for appraising the accuracy of DL model: concordance index, the receiver operating characteristic curve, specificity, sensitivity, precision rate, accuracy, recall rate, calibration curve, F_1_-score, or confusion matrix; and (3) studies published in English.

Exclusion criteria were as follows: (1) conference abstracts without full-text publication; (2) studies limited to traditional machine learning, without the development of DL models; and (3) studies applying DL solely for image segmentation, without developing models for the diagnosis or classification of COPD. Although a very small number of the included studies may have used data from the same public database, we still included these studies because their DL models incorporated comparable experimental designs, which helped us better understand the diagnostic performance of DL models for COPD.

### Data Sources and Search Strategy

The search methods and reporting were guided by PRISMA-S [12]. Embase, Web of Science, the Cochrane Library, and PubMed were systematically searched from database inception to November 1, 2025. The search strategy was designed by combining medical subject headings and free-text keywords. To maximize the retrieval of relevant studies, no restrictions were applied on language or geographic location. The complete search strategies are provided in Table S1 in Multimedia Appendix 1.

We screened the reference lists of the included studies and relevant reviews; we did not search gray literature or conference proceedings and did not contact authors for additional data. No published search filters were used. Search strategies were developed de novo and were not adapted or reused from prior reviews. We did not conduct a formal peer review of the search strategy.

### Study Selection

The retrieved studies were imported into EndNote. Duplicates were automatically and manually removed. Subsequently, the titles and abstracts of the remaining articles were independently reviewed by 2 authors (YH and YW) to identify potentially eligible studies. The full texts of these studies were then assessed to identify eligible studies. Any disagreements at any stage were resolved through discussion with a third reviewer (TW).

### Data Extraction

Before data extraction, a standardized extraction form was developed. The collected data encompassed study title, publication year, DOI, country, authors, patient source, study design, task type, COPD diagnostic criteria, imaging modality used for modeling, number of COPD cases, total number of cases, number of COPD cases in the training set, total number of cases in the training set, method for validation set generation, external validation, number of COPD cases in the validation set, total number of cases in the validation set, and comparison with clinicians (yes or no). Two reviewers (HY and TW) independently extracted the data, followed by cross-checking. Any disagreements were addressed through consultation with a third reviewer (YW).

### Risk of Bias in Studies

The QUADAS-2 (Quality Assessment of Diagnostic Accuracy Studies-2) tool was utilized to appraise the risk of bias (RoB) of the selected studies. The assessment covered 4 domains: reference standard, index test, patient selection, as well as flow and timing. Each domain included several specific questions, which were answered by “Yes (low RoB),” “No (high RoB),” or “Unclear (RoB uncertain).” The overall RoB for each domain was categorized as low, high, or unclear. The RoB assessment was independently performed by 2 reviewers (YW and TW), and disagreements were addressed through discussion with a third reviewer (HY).

### Synthesis Methods

For binary classification tasks, a bivariate mixed effects model was used to pool diagnostic 2×2 contingency tables for DL for the diagnosis of COPD. In studies without complete contingency tables, specificity, sensitivity, negative and positive predictive values, accuracy, and the number of cases were used to estimate the contingency table. Sensitivity, specificity, negative likelihood ratio (NLR), positive likelihood ratio (PLR), diagnostic odds ratio (DOR), and the summary receiver operating characteristic curve with corresponding 95% CIs were pooled. Deeks’ funnel plot was applied to examine the small-study effects of the selected original studies, and clinical applicability was assessed through nomograms. Subgroup analyses by modality (CT, respiratory sounds, or CXR) were performed. All meta-analyses were based on validation set data. If a study reported multiple validation cohorts, each independent validation cohort was included in the analysis separately. If multiple models were evaluated on the same validation cohort, only 1 estimate (ie, the primary and final model reported) was extracted to avoid the nonindependence of the data.

For multiclass classification tasks, the accuracy across different severity grades was pooled. When the reported accuracy approached 99%, a double arcsine transformation was applied before meta-analysis. During the meta-analysis, we utilized the Hartung-Knapp-Sidik-Jonkman modified method [15]. Due to the potential heterogeneity, the 95% prediction intervals for the summary estimates were calculated using the confidence distribution approach proposed by Nagashima et al [16]. All analyses were carried out using STATA (version 15.0; StataCorp LLC) or R (version 4.4.3; R Foundation for Statistical Computing).

## Results

### Study Selection

**Figure 1.**
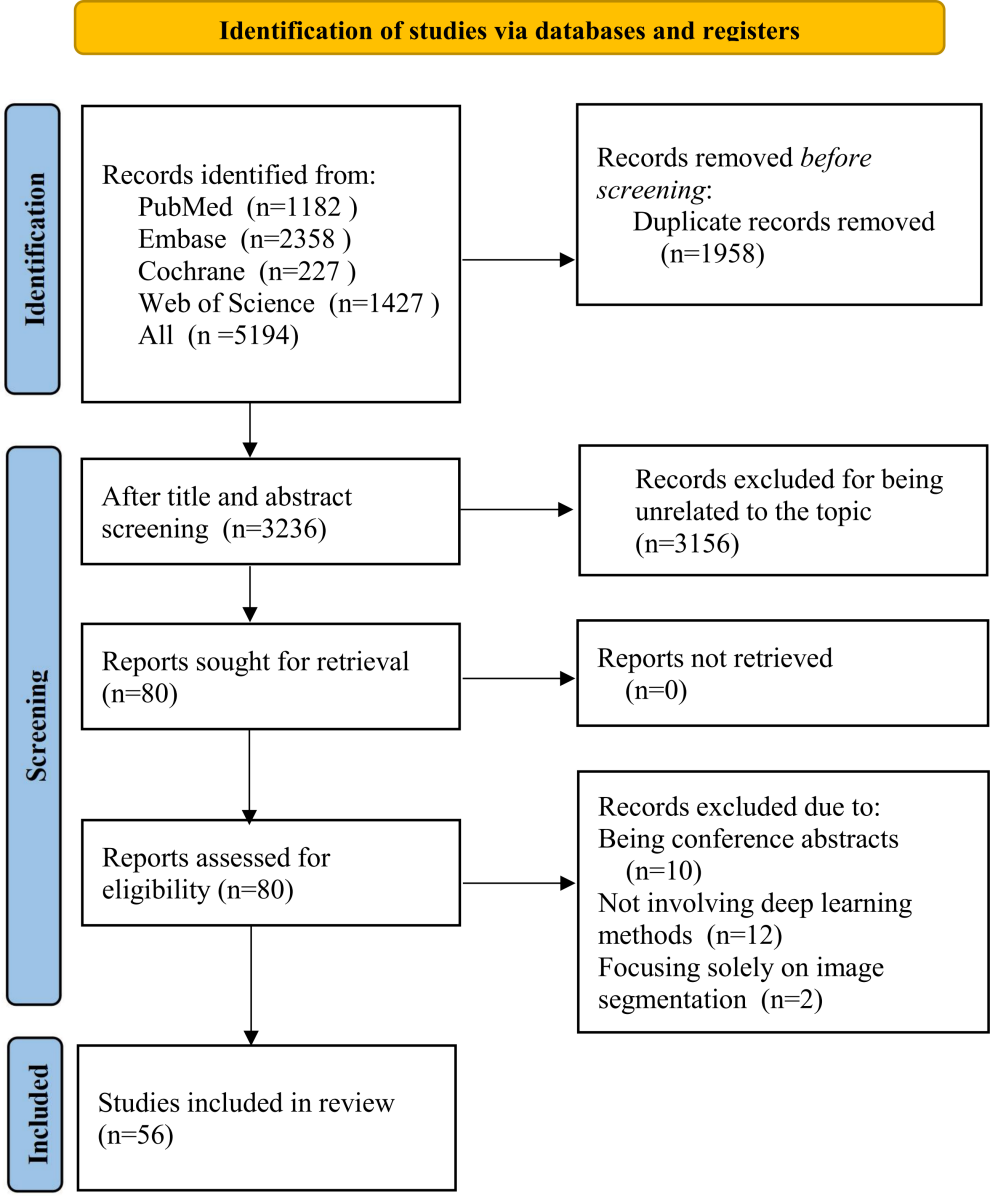
The PRISMA (Preferred Reporting Items for Systematic Reviews and Meta-Analyses) flowchart for the systematic review and diagnostic test accuracy meta-analysis, showing the entire process from the database search in Embase, Web of Science, Cochrane Library, and PubMed up to November 1, 2025, to the literature screening and final inclusion of 56 studies on chronic obstructive pulmonary disease (COPD) in adults.

Overall, 5194 records were retrieved from databases. After excluding 1958 duplicates, we removed 1695 studies unrelated to the study topic and 492 studies for other reasons. The titles and abstracts of 1049 studies were checked. Among them, 969 studies were removed due to irrelevant or unsuitable study design. The full texts of 80 articles were assessed for eligibility, among which 24 ineligible studies were further excluded. Ultimately, 56 studies [10,17-71] were included (Figure 1).

### Study Characteristics

The 56 selected studies were published between 2019 and 2025 across 14 countries, with the majority conducted in China (n=21) and the United States (n=11). In terms of study design, there were 39 cohort studies (including retrospective cohort studies), 16 case-control studies, and 1 retrospective cross-sectional diagnostic study. Most datasets were derived from single-center (n=16) or multi-center (n=31) studies, while 9 studies utilized registry databases. Regarding task types, 23 studies focused solely on diagnosis, 17 studies solely on classification, and 16 studies on both diagnosis and classification (out of 16). All studies clearly reported the diagnostic criteria for COPD. The variables of the models primarily came from CT images (30 studies) and breath sound or audio data (12 studies); 4 studies used CXRs (including X-ray films in 2 studies); the remaining 10 studies used other input data (eg, pulmonary function indicators or curves or waveforms, electrocardiograms, volumetric carbon dioxide monitoring, clinical data, imaging-genetic data, or CT-based scores). The total number of cases was 886,753, with 272,881 in the validation sets and 1,352,782 in the training sets. The methods for generating the validation set were categorized as follows: only cross-validation used in 22 studies; only internal validation in 20 studies; external validation in 9 studies; a combination of internal and external validation in 3 studies; and a combination of cross-validation, internal validation, and external validation in 1 study. One study did not report its validation strategy (1 study; Table S2 in Multimedia Appendix 1).

### RoB in Studies

**Figure 2.**
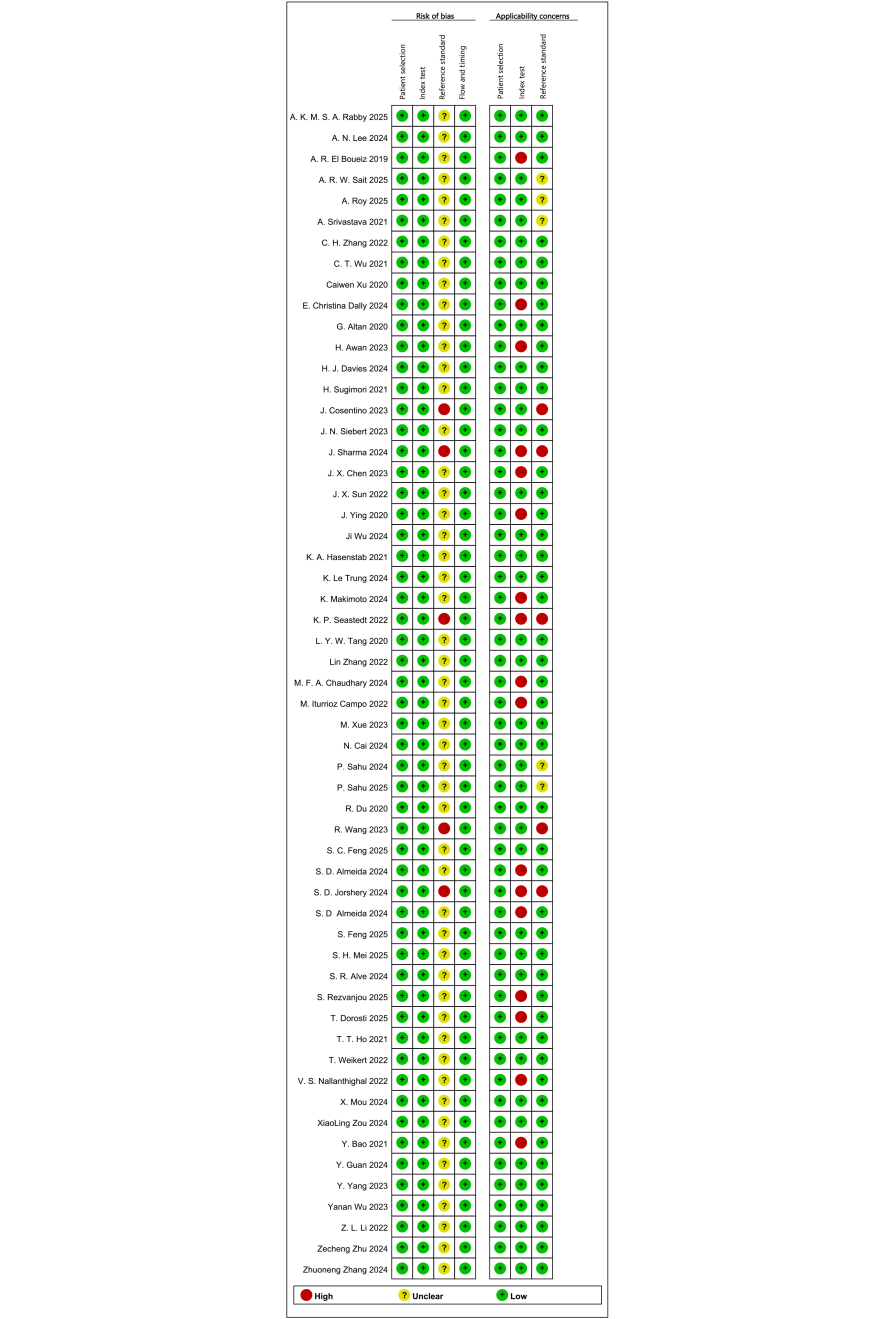
Detailed QUADAS-2 (Quality Assessment of Diagnostic Accuracy Studies-2) risk-of-bias assessment process for the included 56 diagnostic accuracy studies on deep learning (DL) models for chronic obstructive pulmonary disease (COPD) [10,17-19,21,23,24,26-29,31-35,37-41,43-61,63-70,72].

In the patient selection domain, all studies employed consecutive or random case selection and applied appropriate exclusion criteria, thereby avoiding including inappropriate cases; therefore, RoB was judged to be low in this domain. For the index test domain, the included studies generally applied supervised DL methods with clearly defined decision rules, and RoB was judged to be mostly low. Regarding the reference standard, all studies used appropriate diagnostic criteria capable of effectively distinguishing COPD and its severity; however, if a study did not explicitly report whether the reference standard assessment was performed blinded to the index test, we rated this item as unclear, leading to an overall judgment of unclear RoB in the reference standard domain for those studies. For the flow and timing domain, RoB was generally low, although incomplete reporting of participant flow and timing resulted in some unclear judgments. In terms of applicability, patient selection was largely consistent with the review question, while a subset of studies raised applicability concerns related to the index test, the reference standard, or both. In addition, some studies reported only summary performance metrics (eg, accuracy) without complete 2×2 contingency tables, which limited transparency for evidence synthesis and introduced uncertainty when reconstructing contingency tables (Figures2  3).

**Figure 3.**
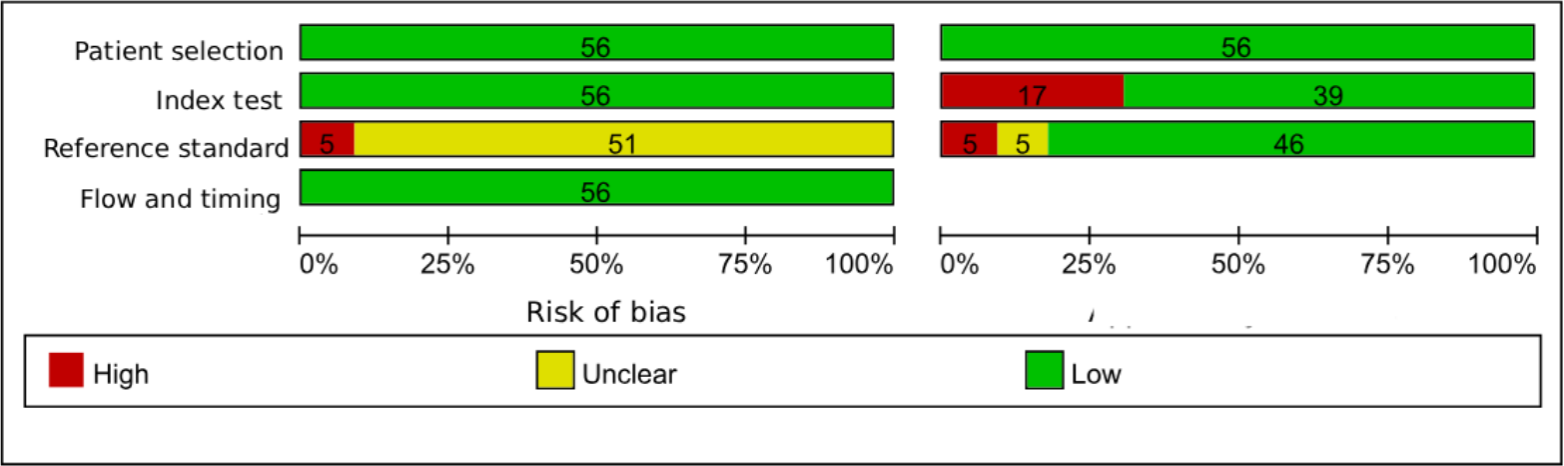
Summary of QUADAS-2 (Quality Assessment of Diagnostic Accuracy Studies-2) risk-of-bias and applicability assessments for the 56 diagnostic accuracy studies on deep learning (DL) models for chronic obstructive pulmonary disease (COPD).

### Meta-Analysis of Binary Classification Tasks

#### Overall

**Figure 4.**
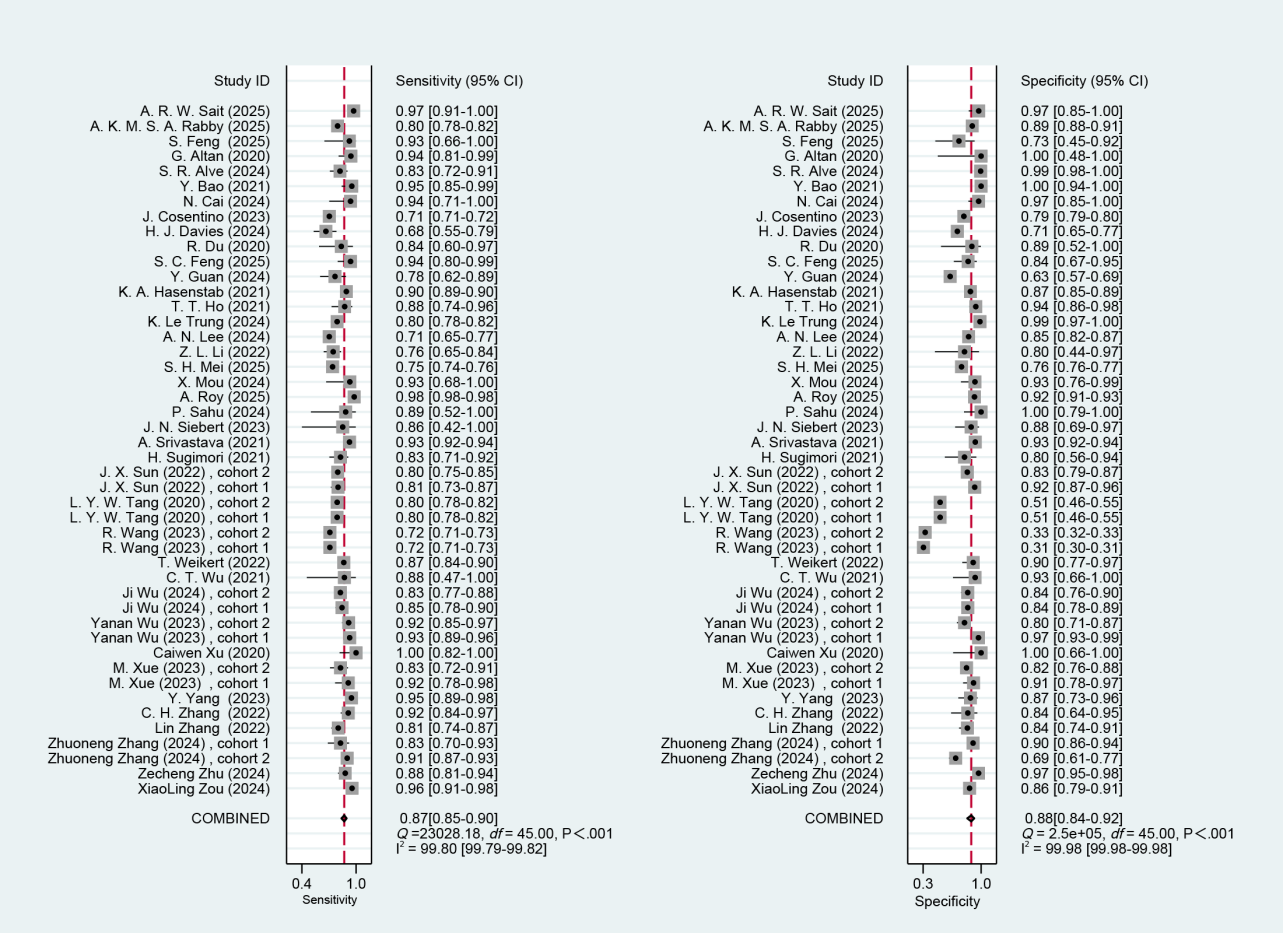
Forest plots of sensitivity and specificity of deep learning (DL) model for binary classification diagnosis of chronic obstructive pulmonary disease (COPD), summarizing 2×2 contingency table results from 43 validation cohorts in 14 countries from 2019 to 2025 [10,11,17,18,20,22,23,25-34,38,40,41,43-45,48-51,53,54,56,61,63,67,68,70,73].

A total of 43 diagnostic 2×2 contingency tables were synthesized to appraise the diagnostic accuracy of DL models for COPD. The pooled results demonstrated that the DL models yielded a sensitivity of 0.87 (95% CI: 0.85‐0.90), specificity of 0.88 (95% CI 0.84‐0.92), PLR of 7.4 (95% CI 5.2‐10.5), NLR of 0.14 (95% CI 0.11‐0.18), DOR of 52 (95% CI 30‐88), and the area under the summary receiver operating characteristic curve (AUC) of 0.93 (95% CI 0.18‐1.00; Figures4  5). Deeks’ funnel plot demonstrated no significant small-study effects (*P*=.08; Figure 6). Assuming a pretest probability of 25%, the posttest probability rose to about 71% for a positive result and decreased to about 5% for a negative result, suggesting the potential clinical value of the models in the screening and diagnosis of COPD (Figure 7).

**Figure 5.**
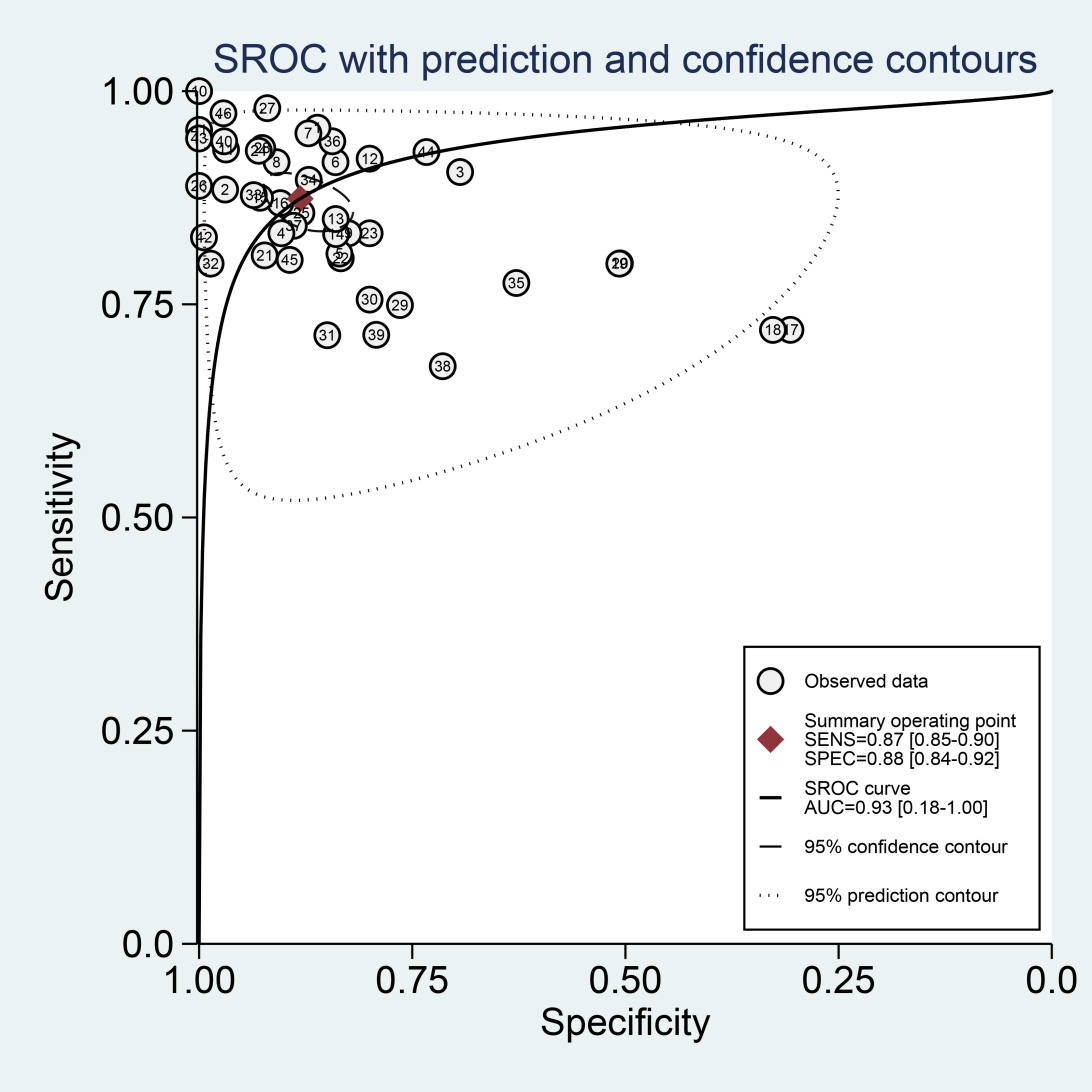
Summary receiver operating characteristic (SROC) curve for the meta-analysis of deep learning (DL) for the diagnosis of chronic obstructive pulmonary disease (COPD) in the validation sets. AUC: area under the summary receiver operating characteristic curve; SENS: sensitivity; SPEC: specificity.

**Figure 6.**
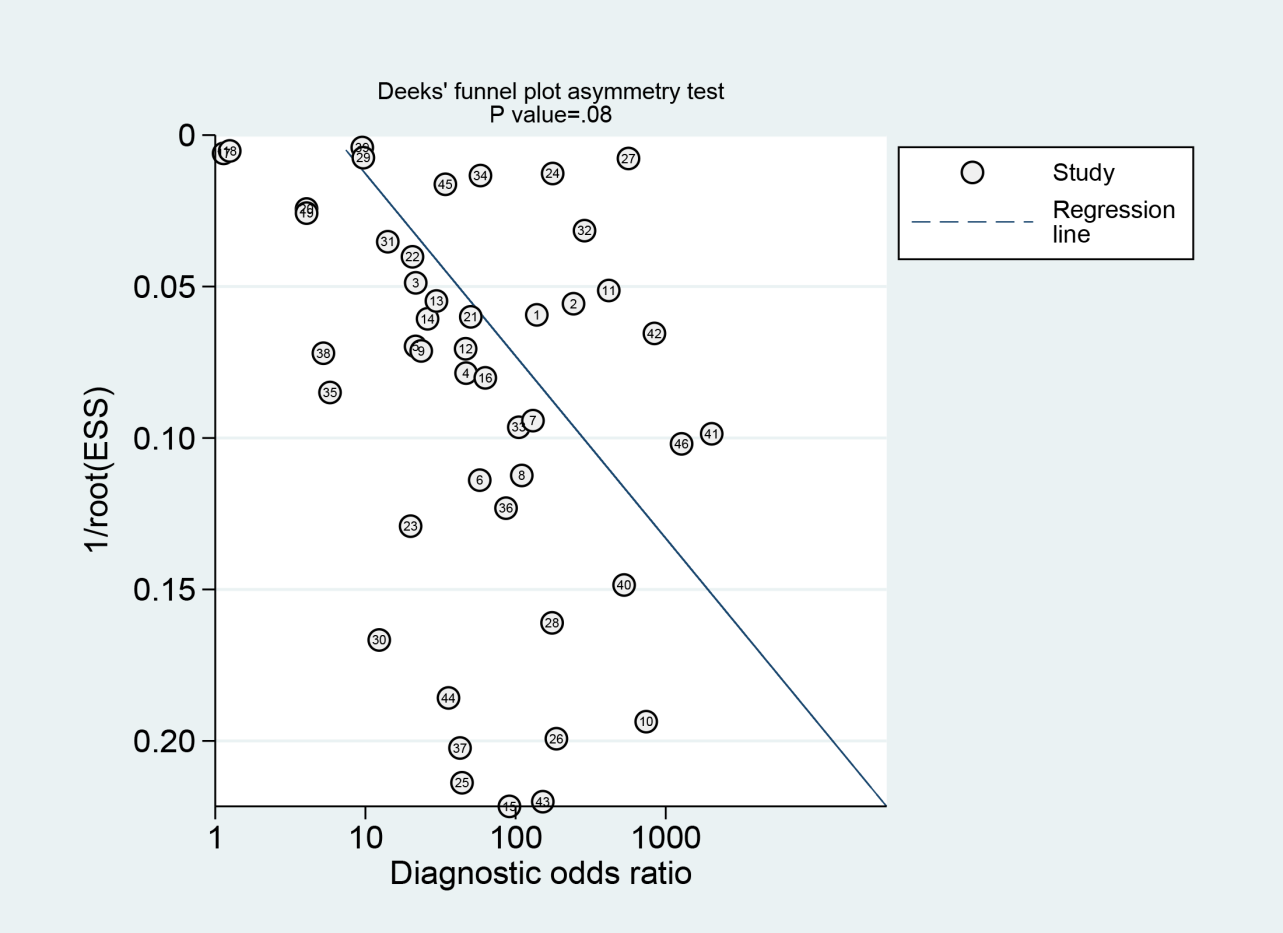
Deeks funnel plot of deep learning (DL) model in the binary classification diagnosis of chronic obstructive pulmonary disease (COPD), assessing publication bias and small-sample effect based on 43 validation cohorts. ESS: effective sample size.

**Figure 7.**
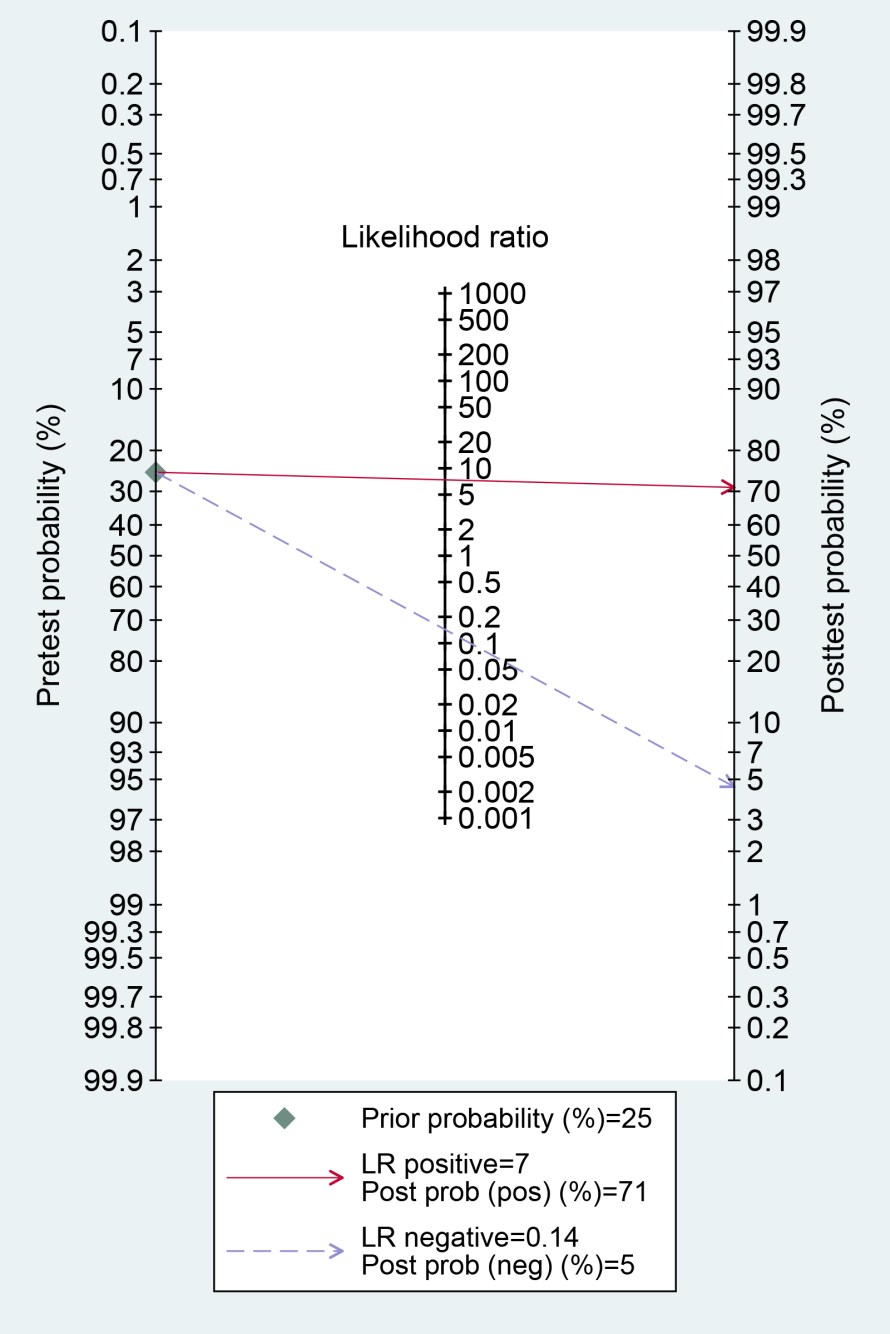
The Fagan cursor plot for deep learning (DL) model in the binary classification diagnosis of chronic obstructive pulmonary disease (COPD), providing the posterior probabilities of positive and negative results with a 25% predetection probability in individuals with suspected COPD based on the pooled likelihood ratio (43 validation cohorts).

#### DL Based on CT Images

A total of 30 contingency tables were included. The pooled sensitivity of the models was 0.86 (95% CI 0.84‐0.89), specificity was 0.87 (95% CI 0.82‐0.90), PLR was 6.6 (95% CI 4.8‐9.1), NLR was 0.15 (95% CI 0.12‐0.19), DOR was 42 (95% CI 26‐68), and AUC was 0.92 (95% CI 0.90‐0.94; Figures S1-S2 in Multimedia Appendix 1). Deeks’ test indicated potential small-study effects (*P*=.02; Figure S3 in Multimedia Appendix 1). Assuming a pretest probability of 25%, the posttest probability rose to 69% for a positive result and decreased to 5% for a negative result (Figure S4 in Multimedia Appendix 1).

Among these, 24 contingency tables were derived from internal validation sets. The pooled sensitivity was 0.86 (95% CI 0.83‐0.89), specificity was 0.88 (95% CI 0.83‐0.92), PLR was 7.4 (95% CI 5.0‐10.9), NLR was 0.16 (95% CI 0.12‐0.20), DOR was 48 (95% CI 26‐86), and AUC was 0.93 (95% CI 0.90‐0.95; Figures S5–S6 in Multimedia Appendix 1). Deeks’ funnel plot demonstrated small-study effects (*P*=.04; Figure S7 in Multimedia Appendix 1). Assuming a pretest probability of 25%, the posttest probability increased to about 71% for a positive result and decreased to about 5% for a negative result (Figure S8 in Multimedia Appendix 1). Among studies on CT-based DL, most models incorporated lung parenchymal attenuation patterns related to emphysema. Some additionally incorporated airway and bronchial wall morphology; gas trapping and small-airway abnormalities on inspiratory or expiratory CT; or combined radiomics features of lung parenchyma, airways, and pulmonary vessels.

A total of 8 contingency tables originated from external validation sets. The pooled sensitivity was 0.87 (95% CI 0.82‐0.90), specificity was 0.83 (95% CI 0.72‐0.90), PLR was 5.1 (95% CI 2.9‐8.8), NLR was 0.16 (95% CI 0.11‐0.23), DOR was 31 (95% CI 14‐72), and AUC was 0.91 (95% CI 1.00‐0.00; Figures S9-S10 in Multimedia Appendix 1). Deeks’ funnel plot demonstrated no small-study effects (*P*=.06; Figure S11 in Multimedia Appendix 1). Assuming a pretest probability of 25%, the posttest probability rose to about 63% for a positive result and decreased to about 5% for a negative result (Figure S12 in Multimedia Appendix 1).

To further evaluate potential small-study effects, we additionally stratified the CT-based validation cohorts by the number of COPD cases in the validation set. A total of 15 cohorts were classified as a small-sample subgroup (COPD cases <100) and 17 cohorts as a large-sample subgroup (COPD cases ≥100). In the small-sample subgroup, the pooled sensitivity and specificity were 0.89 (95% CI 0.84‐0.92) and 0.89 (95% CI 0.83‐0.94), respectively, with an AUC of 0.94 (95% CI 0.92‐0.96). In the large-sample subgroup, the pooled sensitivity and specificity were slightly lower at 0.85 (95% CI 0.82‐0.88) and 0.85 (95% CI 0.78‐0.90), respectively, with an AUC of 0.91 (95% CI 0.88‐0.93; Figures S13-S16 in Multimedia Appendix 1). Assuming a pretest probability of 25%, the Fagan nomograms indicated that the posttest probability increased to 74% for a positive DL result in the small-sample studies and 65% in the large-sample studies, while it reduced to 4% and 6% for a negative result, respectively (Figures S17 and S18 in Multimedia Appendix 1). Deeks’ funnel plot asymmetry tests for the small- and large-sample subgroups were not statistically significant (*P*=.34 and *P*=.15, respectively; Figures S19 and S20 in Multimedia Appendix 1), suggesting no strong evidence of small-study effects. However, given the consistently higher point estimates in the small-sample subgroup, some degree of small-study effects cannot be completely ruled out.

#### DL Based on Respiratory Sounds

A total of 10 contingency tables were included. The pooled sensitivity was 0.91 (95% CI 0.84‐0.95), specificity was 0.96 (95% CI 0.91‐0.98), PLR was 22.1 (95% CI 9.5‐51.5), NLR was 0.09 (95% CI 0.05‐0.18), DOR was 237 (95% CI 78‐723), and AUC was 0.98 (95% CI 0.96‐0.99; Figures S21-S22 in Multimedia Appendix 1). Deeks’ funnel plot demonstrated no small-study effects (*P*=.32; Figure S23 in Multimedia Appendix 1). With a pretest probability of 25%, the posttest probability rose to about 88% following a positive result and decreased to about 3% following a negative result (Figure S24 in Multimedia Appendix 1). For respiratory sound–based DL models, lung sounds were recorded using electronic or digital stethoscopes at standard chest auscultation sites or obtained from open respiratory sound databases (eg, RespiratoryDatabase@TR and other multichannel lung sound datasets) and analyzed as single- or multichannel signals.

#### Summary of DL Based on CXR

Only 2 included studies evaluated DL models based on CXR for the diagnosis of COPD. In a multicenter study, Zou et al [10] constructed a DL model integrating CXR images and clinical parameters. This model achieved favorable performance in internal validation with a sensitivity of 0.96 and a specificity of 0.86. Conversely, Wang et al [29] constructed a model solely based on CXR images. Their model yielded a sensitivity of 0.72 and specificity of 0.31 in the MIMIC-CXR internal validation set and a sensitivity of 0.72 and specificity of 0.33 in the Emory-CXR external validation set. These findings suggest that combining clinical parameters with imaging data may substantially enhance diagnostic performance, whereas single-image models exhibit limited specificity.

#### Summary of DL Based on Externally Applied Airway Resistance

In the study by Davies [54], a physical simulation device was utilized to generate surrogate data for training a DL model. Tubes of varying diameters (3‐25 mm) were installed in the respiratory tract of healthy participants to independently modulate inspiratory and expiratory resistance, thereby simulating COPD-related obstruction. Based on the generated photoplethysmography signals, a 1D convolutional neural network achieved an AUC of 0.75 in the binary classification of COPD and healthy controls. The accuracy of the model reached 40%‐88% for real COPD cases, with a 14% misdiagnosis rate in healthy participants. This approach may offer a low-cost alternative for data-scarce scenarios, particularly suitable for screening with wearable devices in primary care. However, since dynamic resistance simulation was limited, and the sample size for validation was small (only 4 patients), the model needs to be further optimized.

### Multiclass DL for COPD Grading

A total of 6 studies [10,22,31,32,40,44] developed DL models for GOLD grading of COPD (multiclass classification). Among these studies, 5 developed models based on CT images, while Zou et al [10] used CXR images for modeling. Most studies applied different GOLD classification strategies. Several studies [10,31,40,44] implemented 5-class classification (GOLD 0‐4). In another analysis by Zou [10], a 3-class strategy was applied (GOLD 0, GOLD 1‐2, GOLD 3‐4). Sugimori [32] and Yang [22] employed a 4-class strategy (GOLD 0, 1, 2, 3‐4).

Overall analysis indicated considerable differences in the accuracy of the DL models for identifying each GOLD stage, reflecting substantial heterogeneity in model performance. The pooled results based on a random-effects model were as follows: the diagnostic accuracy was 0.842 (95% CI 0.605‐0.982) for GOLD 0, 0.617 (95% CI 0.407‐0.808) for GOLD 1, 0.679 (95% CI 0.376‐0.917) for GOLD 2, 0.708 (95% CI 0.163‐1.000) for GOLD 3, and 0.708 (95% CI 0.163‐1.000) for GOLD 4 (Figure S25 in Multimedia Appendix 1). These findings demonstrated that the DL models were unstable in the identification of mild (GOLD 1) and very severe (GOLD 4) stages. Given the wide CIs, the diagnostic accuracy was still limited.

## Discussion

### Summary of the Main Findings

Current DL models for detecting COPD are primarily constructed based on CT imaging and respiratory sound data. The tasks are generally divided into binary and multiclass classifications. Our findings suggested that in binary classification tasks, the CT-based models performed well in internal validation cohorts, with a pooled sensitivity of 0.86 (95% CI 0.83‐0.89) and specificity of 0.88 (95% CI 0.83‐0.92). The models based on respiratory sounds yielded a sensitivity of 0.91 (95% CI 0.84‐0.95) and specificity of 0.96 (95% CI 0.91‐0.98), indicating a strong exclusion ability.

In multiclass classification tasks, the included studies mainly focused on the staging of GOLD. Overall analysis demonstrated that the DL models were unstable for discriminating between different GOLD stages. This finding supports our hypothesis that compared with binary diagnosis, the accuracy and reliability of the DL models for staging COPD still need to be improved.

### Comparison With Previous Reviews

Prior studies have examined the application of CT and respiratory sounds in the diagnosis of COPD. The systematic review and network meta-analysis carried out by Balasubramanian et al [74] focuses on the diagnostic performance of CT-guided transthoracic biopsy or fine-needle aspiration in lung diseases, particularly lung cancer. Their study included 363 studies involving 79,519 patients and reported a pooled sensitivity of 88.9% but did not address the use of CT in the diagnosis of COPD. In addition, Arts et al [75] have evaluated the use of respiratory sounds for diagnosing acute pulmonary diseases. Their results demonstrate that respiratory sounds have a sensitivity of 37% (95% CI 30%‐47%) and specificity of 89% (95% CI 85%‐92%) for diagnosing COPD, based on approximately 12 relevant studies [75]. Willer et al [73] have examined the performance of X-ray dark-field imaging in detecting and evaluating emphysema in patients with COPD. Their study includes 77 patients and reports that this imaging modality exhibits high diagnostic performance for emphysema (correlation coefficient ρ=0.62, *P*<.0001) and is closely associated with microstructural changes in the lung. These findings suggest that dark-field chest imaging may be a rapid, low-dose, and sensitive tool for the screening and assessment of COPD. However, their study does not evaluate the diagnostic accuracy of conventional CXR for COPD.

In contrast, this meta-analysis reported higher diagnostic performance of the DL models based on CT imaging and respiratory sounds. The pooled results demonstrated that the DL models based on CT yielded a sensitivity of 0.86 (95% CI 0.84‐0.89) and specificity of 0.87 (95% CI 0.82‐0.90), while respiratory sound–based models yielded a sensitivity of 0.91 (95% CI 0.84‐0.95) and specificity of 0.96 (95% CI 0.91‐0.98). These results suggest that DL approaches might outperform traditional diagnostic methods. Earlier research has also investigated the role of artificial intelligence (AI) in COPD diagnosis. For instance, Wu et al [72] examined the potential of machine learning and DL in the detection, staging, and quantitative analysis of COPD using CT imaging. However, their review does not clearly differentiate between machine learning and DL, nor does it discuss in depth the advantages and limitations of image-based AI models for the diagnosis of COPD.

This study found that the included studies on DL for diagnosing COPD focused mainly on CT imaging, respiratory sounds, CXR, and externally applied airway resistance. Among these, CT, respiratory sounds, and CXR were the most frequently used data sources for model development and carried distinct clinical implications. Chest CT exerts a crucial role in diagnosing and phenotyping COPD, as it can identify structural abnormalities, such as airway narrowing and emphysema, and is recommended by current clinical guidelines. Our findings demonstrated that the CT-based DL models offered excellent specificity and sensitivity for the diagnosis of COPD, suggesting their potential as auxiliary diagnostic tools in clinical practice. The DL models based on respiratory sounds, as a non-invasive and portable modality, also had good diagnostic performance, particularly with high specificity, indicating potential value in primary screening. In contrast, the number of studies using CXR remains limited, and the existing evidence is insufficient to determine the stability and generalizability of CXR-based DL models for diagnosing COPD. It should be validated in the future. Moreover, although a few preliminary studies have explored the use of externally applied airway resistance to generate model inputs, the number of studies remains small, and reproducible, generalizable evidence is lacking. Thus, future studies are required to assess the utility and reliability of this approach in clinical practice.

Despite the promise of AI in the diagnosis of COPD, significant challenges need to be addressed before widespread clinical application, particularly in explainability and data integration. Although current research demonstrates encouraging diagnostic performance, a substantial gap persists between theoretical development and real-world application. First, most included studies did not thoroughly examine how variations in imaging protocols, such as scan parameters or reconstruction algorithms, influence image features and the performance of DL models. Hence, a systematic evaluation of these factors is lacking. Second, as complex neural network frameworks, DL models rely on large-scale training datasets to improve robustness. However, most included studies developed models using limited samples, with only a few utilizing large datasets. The scarcity of data represents a core bottleneck in model development, constraining the generalizability of the models. Future studies should incorporate richer and more diverse imaging data. Third, the current model evaluation primarily relied on internal validation techniques, such as random sampling, cross-validation, or bootstrap methods. While internal validation sets share similar distributions with training data and often yield favorable results, they do not accurately reflect the generalizability of the models on heterogeneous datasets. Models should be rigorously externally validated before real-world application, particularly across institutions and using datasets obtained under different imaging protocols. Studies based on high-quality external validation remain scarce, and substantial differences in imaging protocols make it challenging to interpret model performance in external validation.

In clinical research and practice, grading disease severity is as crucial as diagnosing COPD. The widely applied GOLD classification, which stratifies COPD into 5 grades (0 and 1‐4), reflects significant differences in clinical presentation, treatment strategies, and prognosis of COPD. Achieving early and precise grading is therefore of high clinical relevance. However, only 6 studies have attempted to develop DL models for grading the severity of COPD, providing limited evidence. These studies indicate that DL models generally perform suboptimally in multiclass classification tasks, with particularly low accuracy for GOLD 1, GOLD 2, and GOLD 4. These models achieve relatively higher accuracy only for GOLD 0 and GOLD 3, exceeding 70%. Nevertheless, their stability still needs to be enhanced. This suggests that multiclass classification itself represents a technical challenge for DL models. Moreover, under the current dataset size, label distribution, and model architecture, stable differentiation across all GOLD grades remains difficult. Future research should aim to enhance the discriminative ability of models, incorporate richer imaging data, and integrate clinical information to optimize training strategies, ultimately developing more accurate and adaptable intelligent tools for grading the severity of COPD to support clinical decision-making.

### Strengths and Limitations of the Study

This meta-analysis systematically assessed the performance of DL in the detection of COPD for the first time, providing evidence to support the development of intelligent diagnostic tools. The findings indicate that DL models hold substantial potential for improving diagnostic accuracy, particularly through noninvasive and nonintrusive detection methods. This study provides valuable insights. However, some limitations must be noted. First, although a systematic literature search was carried out, the number of studies focusing on respiratory sounds remained relatively small. As respiratory sound analysis is an emerging diagnostic approach, the number and diversity of relevant studies remain far below those of CT imaging, which may limit a comprehensive assessment of this method. Second, most included studies relied primarily on internal validation, and only relatively few studies performed external validation. Although internal validation can provide some indication of diagnostic accuracy, limitations in sample size and validation methods may compromise the generalizability of the results. To further confirm the clinical utility of DL models, future studies should perform external validation. Third, research on the severity of COPD was relatively scarce, and some studies employed differing grading strategies. These variations may affect the reliability of classification models and the generalizability of their findings. Thus, this finding should be cautiously interpreted.

### Heterogeneity and Clinical Applicability of DL Models

Although subgroup analyses were performed to explore the source of heterogeneity, significant heterogeneity still existed among the subgroups. This heterogeneity may stem from differences in DL frameworks used in different studies, such as 2D or 3D convolutional neural networks, multiview networks, multi-instance learning, and late fusion. The included studies used diverse DL models, which differed in network structure, input format, and parameter settings. Consequently, their model training and validation methods may also differ. Therefore, these differences in structure and parameters can lead to potential heterogeneity, which is a common challenge in current meta-analyses of DL models.

From the perspective of clinical practicality, DL still holds significant advantages over traditional radiomics. Traditional radiomics typically requires manual or semiautomatic image segmentation, followed by the extraction of a limited number of manual features, such as texture. An original image is compressed into a small number of quantitative features, then to a machine learning model. This multistep process is time-consuming, highly dependent on the researcher’s experience, and may lose some original image information during dimensionality reduction and feature selection. DL, on the other hand, can directly train models end-to-end based on labeled (or segmented) images without additional feature engineering. It can preserve lesion-related image information to the greatest extent, potentially improving model performance and reducing manual operations and time costs. Therefore, given the relatively ideal diagnostic and grading accuracy of DL models, it is hoped that AI-assisted diagnostic DL tools should be developed to support, rather than replace, clinicians in screening and assessing the severity of COPD.

### Future Perspectives

Most current studies are based on relatively limited imaging datasets and rely mainly on internal validation. Thus, the reported accuracy may not fully reflect the generalizability of models. Given substantial between-study heterogeneity and limited external validation, these findings should be interpreted cautiously. Future research should improve and update these DL models by using larger, multicenter imaging datasets from different geographical regions and scanners, and by incorporating robust external validation and more rigorous model development strategies.

To our knowledge, this is the first systematic synthesis to quantify the diagnostic and grading performance of DL models across major data sources (eg, CT imaging and respiratory sounds), showing promising accuracy for binary COPD detection but suboptimal and less stable performance for multiclass GOLD staging.

In summary, our comprehensive study on DL provides an evidence base for guiding the development and external validation of AI-assisted screening tools for COPD, especially given the insufficient application of spirometry.

### Conclusions

This study observed that DL models achieved promising accuracy in the detection of COPD. The models performed particularly well in binary classification tasks, exhibiting high sensitivity and specificity. However, its accuracy was suboptimal in multiclass tasks for grading the severity of GOLD. In addition, research on respiratory sound analysis and multiclass classification of COPD severity is still limited. Given the substantial heterogeneity and limited external validation, these results should be interpreted cautiously. Thus, future research should integrate larger and more diverse imaging datasets, particularly including images from different racial populations, to develop more robust and generalizable intelligent diagnostic tools. This approach would not only enhance the generalizability of models but also improve the accuracy of diagnosing COPD across diverse patient groups.

## Supplementary material

10.2196/83459 Multimedia Appendix 1 Supplementary materials (search strategies, study characteristics, and additional analyses).

10.2196/83459 Checklist 1 PRISMA-S checklist.

## Abbreviations

AI: artificial intelligence
AUC: area under the summary receiver operating characteristic curve
COPD: chronic obstructive pulmonary disease
CT: computed tomography
CXR: chest X-ray
DL: deep learning
DOR: diagnostic odds ratio
GOLD: Global Initiative for Chronic Obstructive Lung Disease
NLR: negative likelihood ratio
PFT: pulmonary function testing
PLR: positive likelihood ratio
PRISMA: Preferred Reporting Items for Systematic Reviews and Meta-Analyses
PRISMA-S: Preferred Reporting Items for Systematic reviews and Meta-Analyses literature search extension
PROSPERO: International Prospective Register of Systematic Reviews
QUADAS-2: Quality Assessment of Diagnostic Accuracy Studies-2
RoB: risk of bias
