# Supplementary material for: Accuracy of Deep Learning in Diagnosing Chronic Obstructive Pulmonary Disease: Systematic Review and Meta-Analysis
Source: J Med Internet Res. 2026 Jan 14;28:e83459. doi: 10.2196/83459 (PMC12821088; doi:10.2196/83459)
Supplement: Multimedia Appendix 1 [file jmir-v28-e83459-s001.docx]

Table S1 Detailed search terms for Embase, Web of Science, Cochrane Library, and PubMed, covering observational studies on deep learning in the diagnosis and GOLD grading of COPD from database inception to November 1, 2025, listed according to PRISMA-S

**1.Pubmed**

| Search number | Query | Results |
| --- | --- | --- |
| #1 | "pulmonary disease, chronic obstructive"[MeSH Terms] | 99266 |
| #2 | "pulmonary disease, chronic obstructive"[MeSH Terms] OR "pulmonary disease chronic obstructive"[Title/Abstract] OR "chronic obstructive pulmonary diseases"[Title/Abstract] OR "COPD"[Title/Abstract] OR "chronic obstructive lung disease"[Title/Abstract] OR "chronic obstructive pulmonary disease"[Title/Abstract] OR "COAD"[Title/Abstract] OR "chronic obstructive airway disease"[Title/Abstract] OR "chronic airflow obstructions"[Title/Abstract] OR "chronic airflow obstruction"[Title/Abstract] OR "chronic obstructive lung disease"[Title/Abstract] OR "chronic airflow obstruction"[Title/Abstract] OR "chronic airway obstruction"[Title/Abstract] OR "chronic obstructive bronchopulmonary disease"[Title/Abstract] OR "chronic obstructive lung disorder"[Title/Abstract] OR "chronic obstructive pulmonary disease"[Title/Abstract] OR "chronic obstructive pulmonary disorder"[Title/Abstract] OR "chronic obstructive respiratory disease"[Title/Abstract] OR "chronic pulmonary obstructive disease"[Title/Abstract] OR "chronic pulmonary obstructive disorder"[Title/Abstract] OR (("Lung"[MeSH Terms] OR "Lung"[All Fields]) AND "chronic obstructive disease"[Title/Abstract]) OR "obstructive chronic lung disease"[Title/Abstract] OR "obstructive chronic pulmonary disease"[Title/Abstract] | 135476 |
| #3 | "Deep Learning"[MeSH Terms] | 27204 |
| #4 | "Deep Learning"[MeSH Terms] OR "Deep Learning"[Title/Abstract] OR "transfer learning"[Title/Abstract] OR "ensemble learning"[Title/Abstract] OR "artificial intelligence"[Title/Abstract] OR "hierarchical learning"[Title/Abstract] OR "machine learning"[Title/Abstract] OR "neural networks"[Title/Abstract] OR "neural network"[Title/Abstract] OR "deep networks"[Title/Abstract] OR "deep network"[Title/Abstract] OR "Transformer"[Title/Abstract] OR "long short term memory"[Title/Abstract] OR "generative adversarial networks"[Title/Abstract] OR "LSTM"[Title/Abstract] OR "GNN"[Title/Abstract] OR "CNN"[Title/Abstract] OR "RNN"[Title/Abstract] OR "AlexNet"[Title/Abstract] OR "VGGNet"[Title/Abstract] OR "ResNet"[Title/Abstract] OR "GoogLeNet"[Title/Abstract] OR "VGG-11"[Title/Abstract] OR "VGG-13"[Title/Abstract] OR "VGG-16"[Title/Abstract] OR "VGG-19"[Title/Abstract] OR "VGG11"[Title/Abstract] OR "VGG13"[Title/Abstract] OR "VGG16"[Title/Abstract] OR "VGG19"[Title/Abstract] OR "ResNet50"[Title/Abstract] OR "ResNet101"[Title/Abstract] OR "ResNet34"[Title/Abstract] OR "ResNet18"[Title/Abstract] | 400567 |
| #5 | ("pulmonary disease, chronic obstructive"[MeSH Terms] OR "pulmonary disease chronic obstructive"[Title/Abstract] OR "chronic obstructive pulmonary diseases"[Title/Abstract] OR "COPD"[Title/Abstract] OR "chronic obstructive lung disease"[Title/Abstract] OR "chronic obstructive pulmonary disease"[Title/Abstract] OR "COAD"[Title/Abstract] OR "chronic obstructive airway disease"[Title/Abstract] OR "chronic airflow obstructions"[Title/Abstract] OR "chronic airflow obstruction"[Title/Abstract] OR "chronic obstructive lung disease"[Title/Abstract] OR "chronic airflow obstruction"[Title/Abstract] OR "chronic airway obstruction"[Title/Abstract] OR "chronic obstructive bronchopulmonary disease"[Title/Abstract] OR "chronic obstructive lung disorder"[Title/Abstract] OR "chronic obstructive pulmonary disease"[Title/Abstract] OR "chronic obstructive pulmonary disorder"[Title/Abstract] OR "chronic obstructive respiratory disease"[Title/Abstract] OR "chronic pulmonary obstructive disease"[Title/Abstract] OR "chronic pulmonary obstructive disorder"[Title/Abstract] OR (("Lung"[MeSH Terms] OR "Lung"[All Fields]) AND "chronic obstructive disease"[Title/Abstract]) OR "obstructive chronic lung disease"[Title/Abstract] OR "obstructive chronic pulmonary disease"[Title/Abstract]) AND ("Deep Learning"[MeSH Terms] OR "Deep Learning"[Title/Abstract] OR "transfer learning"[Title/Abstract] OR "ensemble learning"[Title/Abstract] OR "artificial intelligence"[Title/Abstract] OR "hierarchical learning"[Title/Abstract] OR "machine learning"[Title/Abstract] OR "neural networks"[Title/Abstract] OR "neural network"[Title/Abstract] OR "deep networks"[Title/Abstract] OR "deep network"[Title/Abstract] OR "Transformer"[Title/Abstract] OR "long short term memory"[Title/Abstract] OR "generative adversarial networks"[Title/Abstract] OR "LSTM"[Title/Abstract] OR "GNN"[Title/Abstract] OR "CNN"[Title/Abstract] OR "RNN"[Title/Abstract] OR "AlexNet"[Title/Abstract] OR "VGGNet"[Title/Abstract] OR "ResNet"[Title/Abstract] OR "GoogLeNet"[Title/Abstract] OR "VGG-11"[Title/Abstract] OR "VGG-13"[Title/Abstract] OR "VGG-16"[Title/Abstract] OR "VGG-19"[Title/Abstract] OR "VGG11"[Title/Abstract] OR "VGG13"[Title/Abstract] OR "VGG16"[Title/Abstract] OR "VGG19"[Title/Abstract] OR "ResNet50"[Title/Abstract] OR "ResNet101"[Title/Abstract] OR "ResNet34"[Title/Abstract] OR "ResNet18"[Title/Abstract]) | 1182 |

**2.Cochrane**

| Search number | Query | Results |
| --- | --- | --- |
| #1 | MeSH descriptor: [Pulmonary Disease, Chronic Obstructive] explode all trees | 8246 |
| #2 | (Chronic Obstructive Pulmonary Diseases or COPD or Chronic Obstructive Lung Disease or Chronic Obstructive Pulmonary Disease or COAD or Chronic Obstructive Airway Disease or Chronic Airflow Obstructions or Chronic Airflow Obstruction or chronic obstructive lung disease or chronic airflow obstruction or chronic airway obstruction or chronic obstructive bronchopulmonary disease or chronic obstructive lung disorder or chronic obstructive pulmonary disease or chronic obstructive pulmonary disorder or chronic obstructive respiratory disease or chronic pulmonary obstructive disease or chronic pulmonary obstructive disorder or lung chronic obstructive disease or obstructive chronic lung disease or obstructive chronic pulmonary disease):ti,ab,kw (Word variations have been searched) | 26358 |
| #3 | #1 or #2 | 27134 |
| #4 | MeSH descriptor: [Deep Learning] explode all trees | 401 |
| #5 | (Transfer Learning or Ensemble Learning or artificial intelligence or Hierarchical Learning or Machine Learning or Neural Networks or Neural Network or Deep networks or Deep network or Transformer or Long Short-Term Memory or Generative Adversarial Networks or LSTM or GNN or CNN or RNN or AlexNet or VGGNet or ResNet or GoogLeNet or VGG-11 or VGG-13 or VGG-16 or VGG-19 or VGG11 or VGG13 or VGG16 or VGG19 or ResNet50 or ResNet101 or ResNet34 or ResNet18):ti,ab,kw (Word variations have been searched) | 26300 |
| #6 | #4 or #5 | 27138 |
| #7 | #3 and #6 | 227 |

**3.Embase**

| Search number | Query | Results |
| --- | --- | --- |
| #1 | 'chronic obstructive lung disease'/exp | 232,055 |
| #2 | 'pulmonary disease, chronic obstructive':ab,ti OR  'chronic obstructive pulmonary diseases':ab,ti OR  copd:ab,ti OR coad:ab,ti OR 'chronic obstructive  airway disease':ab,ti OR 'chronic airflow  obstructions':ab,ti OR 'chronic obstructive lung  disease':ab,ti OR 'chronic airflow  obstruction':ab,ti OR 'chronic airway  obstruction':ab,ti OR 'chronic obstructive  bronchopulmonary disease':ab,ti OR 'chronic  obstructive lung disorder':ab,ti OR 'chronic  obstructive pulmonary disease':ab,ti OR 'chronic  obstructive pulmonary disorder':ab,ti OR 'chronic  obstructive respiratory disease':ab,ti OR  'chronic pulmonary obstructive disease':ab,ti OR  'chronic pulmonary obstructive disorder':ab,ti OR  'lung chronic obstructive disease':ab,ti OR  'obstructive chronic lung disease':ab,ti OR  'obstructive chronic pulmonary disease':ab,ti | 223,546 |
| #3 | #1 OR #2 | 256,000 |
| #4 | 'deep learning'/exp | 98,842 |
| #5 | 'deep learning':ab,ti OR 'transfer  learning':ab,ti OR 'ensemble learning':ab,ti OR  'artificial intelligence':ab,ti OR 'hierarchical  learning':ab,ti OR 'machine learning':ab,ti OR  'neural networks':ab,ti OR 'neural network':ab,ti  OR 'deep networks':ab,ti OR 'deep network':ab,ti  OR transformer:ab,ti OR 'long short-term  memory':ab,ti OR 'generative adversarial  networks':ab,ti OR lstm:ab,ti OR gnn:ab,ti OR  cnn:ab,ti OR rnn:ab,ti OR alexnet:ab,ti OR  vggnet:ab,ti OR resnet:ab,ti OR googlenet:ab,ti  OR 'vgg 11':ab,ti OR 'vgg 13':ab,ti OR 'vgg  16':ab,ti OR 'vgg 19':ab,ti OR vgg11:ab,ti OR  vgg13:ab,ti OR vgg16:ab,ti OR vgg19:ab,ti OR  resnet50:ab,ti OR resnet101:ab,ti OR  resnet34:ab,ti OR resnet18:ab,ti | 432,714 |
| #6 | #4 OR #5 | 463,584 |
| #7 | #3 AND #6 | 2358 |

**4.Web of science**

| Search number | Query | Results |
| --- | --- | --- |
| #1 | (TS=(Pulmonary Disease, Chronic Obstructive) OR AB=(Pulmonary Disease, Chronic Obstructive OR Chronic Obstructive Pulmonary Diseases OR COPD OR Chronic Obstructive Lung Disease OR Chronic Obstructive Pulmonary Disease OR COAD OR Chronic Obstructive Airway Disease OR Chronic Airflow Obstructions OR Chronic Airflow Obstruction OR chronic obstructive lung disease OR chronic airflow obstruction OR chronic airway obstruction OR chronic obstructive bronchopulmonary disease OR chronic obstructive lung disorder OR chronic obstructive pulmonary disease OR chronic obstructive pulmonary disorder OR chronic obstructive respiratory disease OR chronic pulmonary obstructive disease OR chronic pulmonary obstructive disorder OR lung chronic obstructive disease OR obstructive chronic lung disease OR obstructive chronic pulmonary disease)) AND (TS=(Deep Learning) OR AB=(deep learning OR Transfer Learning OR Ensemble Learning OR artificial intelligence OR Hierarchical Learning OR Machine Learning OR Neural Networks OR Neural Network OR Deep networks OR Deep network OR Transformer OR Long Short-Term Memory OR Generative Adversarial Networks OR LSTM OR GNN OR CNN OR RNN OR AlexNet OR VGGNet OR ResNet OR GoogLeNet OR VGG-11 OR VGG-13 OR VGG-16 OR VGG-19 OR VGG11 OR VGG13 OR VGG16 OR VGG19 OR ResNet50 OR ResNet101 OR ResNet34 OR ResNet18)) | 1427 |

Table S2 The baseline characteristics of the 56 included studies, including publication year, country (14 countries, mainly China and the United States), patient source (single-center, multi-center, or registry database), study design, task type (diagnosis/grading), diagnostic criteria for COPD, input modality for DL (CT, chest X-ray, breath sounds), training and validation sample size, validation method, and whether it was compared with clinicians

| First author | Year of publication | Country of author | Study type | Source of patients | Task type | Modeling variables | Number of events (P/I) | Total number of cases (P/I) | Total number of cases in the training set (P/I) | The generation method of the validation set | The number of cases in the validation set |
| --- | --- | --- | --- | --- | --- | --- | --- | --- | --- | --- | --- |
| A. R. El Boueiz | 2019 | United States | Cohort study | Multicenter | Diagnosis | CT images |  | 3,899 | 1,950 | 10-fold cross-validation | 1,950 |
| J. Ying | 2020 | China | Cohort study | Registry database | Classification | CT images |  | 10300 | 9000 | 10-fold cross-validation | 1030 |
| Caiwen Xu | 2020 | China | Case-control study | Multicenter | Classification | CT images | 190 | 280 | 252 | 10-fold cross-validation | 28 |
| L. Y. W. Tang | 2020 | Canada | Cohort study | Multicenter | Classification and diagnosis | CT images | 3014 | 4784 | 2589 | 3-fold cross-validation | 2195 |
| R. Du, | 2020 | China | Case-control study | Single center | Diagnosis | CT images | 190 | 280 | 224 | 10-fold cross-validation | 28 |
| G. Altan | 2020 | Turkey | Case-control study | Single center | Classification and diagnosis | Breath sounds | COPD0: 5 patients, COPD1: 5 patients, COPD2: 7 patients, COPD3: 7 patients, COPD4: 17 patients | 41 | 29-33 | 10-fold cross-validation | 8 |
| C. T. Wu | 2021 | China, Taiwan | Cohort study | Single center | Diagnosis | Breath sounds | 25 | 67 | 22 | 3-fold cross-validation | 22 |
| H. Sugimori | 2021 | Japan | Cohort study | Single center | Classification | CT images | 60 | 80 |  | 4-fold cross-validation | 6500 |
| A. Srivastava | 2021 | India | Case-control study | Multicenter | Classification | Breath sounds | Crackles: 1,864；Wheezes: 886；Both: 506 | 920 audio samples from 126 subjects |  | 10-fold cross-validation |  |
| T. T. Ho | 2021 | Korea | Cohort study | Multicenter | Classification | CT images | 204 | 596 | 477 | 5-fold cross-validation | 119 |
| K. A. Hasenstab | 2021 | United States | Cohort study | Multicenter | Classification | CT images |  |  | 888 | 5-fold cross-validation | 8,951 |
| Y. Bao | 2021 | U.K | Case-control study | Single center | Classification and diagnosis | CT images | 13 | 32 | 26 | Internal validation | 6 |
| [Lin Zhang](https://pubmed-ncbi-nlm-nih-gov-443.ff.sjuku.top/?term=Zhang+L&cauthor_id=35143286) | 2022 | China | Cohort study | Multicenter | Classification | CT images |  | 599 | 373 | External validation | 226 |
| C. H. Zhang | 2022 | China | Cohort study | Single center | Diagnosis | CT images | 84 | 980 | 109 | Internal validation |  |
| T. Weikert | 2022 | Switzerland | Cohort study | Single center | Classification and diagnosis | CT images | 533 | 575 | 460 | Internal validation | 20 |
| J. X. Sun, | 2022 | China | Cohort study | Multicenter | Classification and diagnosis | CT images | 749 | 1393 | 837 | Internal validation | 278 |
| K. P. Seastedt | 2022 | United States | Cohort study | Registry database | Diagnosis | X films | 4,584 | 53,053 | 42,442 | Internal validation | 5,305 |
| V. S. Nallanthighal | 2022 | Netherlands | Case-control study | Single center | Classification | Breath sounds | 11 | 16 | 8 | Internal validation | 3 |
| Z. L. Li | 2022 | China | Cohort study | Registry database | Classification | CT images | 300 | 600 | 400 | Internal validation | 100 |
| M. Iturrioz Campo | 2022 | United States | Cohort study | Multicenter | Classification and diagnosis | CT images | 2,000 | 4,081 | 3,264 | Internal validation | 816 |
| Y. Yang | 2023 | China | Cohort study | Multicenter | Classification | CT images | 465 | 465 | 325 | Random sampling | 140 |
| M. Xue | 2023 | China | Case-control study | Multicenter | Classification | CT images | 363 | 800 | 720 | 10-fold cross-validation | Internal validation set: 80; external validation set: 260 |
| Yanan Wu | 2023 | China | Case-control study | Multicenter | Classification | CT images | 363 | 800 | 720 | 10-fold cross-validation | 80 for validation set (dataset 1) 380 for test set (dataset 2) 201 |
| R. Wang | 2023 | China, Taiwan | Cohort study | Multicenter | Classification and diagnosis | CXR images | MIMIC: 8105 Emory: 10,353 | MIMIC: 52,804 Emory: 10,353 | 203,485 | External validation | 36,453 imaging examinations (MIMIC)+ 14,468 patients (Emory) |
| J. N. Siebert | 2023 | Switzerland | Case-control study | Single center | Classification and diagnosis | Breath sounds | 40 | 160 | 160 | 5-fold cross-validation | 32 |
| J. Cosentino | 2023 | United States | Case-control study | Registry database | Diagnosis | Breath sounds |  | 325,027 | 260,022 | Internal validation | 65,006 |
| J. X. Chen | 2023 | United States | Cohort study | Multicenter | Classification and diagnosis | Imaging data and genetic data |  | 1223 | 923 | Internal validation | 300 |
| H. Awan | 2023 | United States | Cohort study | Multicenter | Diagnosis | CT images | 331 | 1531 | 1224 | 5-fold cross-validation | 307 |
| XiaoLing Zou | 2024 | China | Cohort study | Multicenter | Classification and diagnosis | CXR images | 535 | 1055 | 666 | External validation | 284 |
| Zecheng Zhu | 2024 | China | Cohort study | Single center | Diagnosis | CT images | 497 | 2983 | 1853 | Internal validation | 464 |
| Zhuoneng Zhang | 2024 | China | Cohort study | Multicenter | Diagnosis | CT images | 908 | 2047 | 1224 | External validation | 823 |
| Ji Wu | 2024 | China | Cohort study | Multicenter | Diagnosis | CT images | 90 | 1150 | 519 | External validation | 285 |
| J. Sharma | 2024 | United States | Case-control study | Multicenter | Diagnosis | ECG | 19592 | 78368 | 489,356 | External validation | 106,996 |
| P. Sahu | 2024 | India | Cohort study | Multicenter | Classification and diagnosis | Breath sounds | 45 | 75+126 | 101or 60 | Internal validation | 25 or 15 |
| X. Mou | 2024 | China | Case-control study | Single center | Classification and diagnosis | Volumetric carbon dioxide diagram | 148 | 427 | 384 | 10-fold cross-validation | 42/fold |
| K. Makimoto | 2024 | Canada | Cohort study | Multicenter | Classification | CT images |  | 1,154 | 865 | Internal validation | 289 |
| A. N. Lee | 2024 | United States | Cohort study | Registry database | Classification and diagnosis | CT images | GOLD 0 stage: 3854 patients, GOLD 1 stage: 694 patients, GOLD 2 stage: 1723 patients, GOLD 3 stage: 1022 patients, GOLD 4stage: 530 patients | 8893 | 6820 | Internal validation | 933 |
| K. Le Trung | 2024 | Vietnam | Case-control study | Registry database | Classification and diagnosis | Breath sounds | 2156 | 3636 | 2182 | Internal validation | 727 |
| S. D. Jorshery | 2024 | United States | Cohort study | Multicenter | Diagnosis | X films | 1562(former smokers)+580(never smoking) | Smoking group: 12,550; never smoking group:15,298 | 22,278 | External validation (Multicenter) | Smoking group: 12,550; never smoking group:15,298 |
| Y. Guan | 2024 | China | Cohort study | Single center | Classification | CT images | 169 | 1,024 | 726 | Internal validation | 104 |
| H. J. Davies | 2024 | U.K | Cohort study | Multicenter | Classification | Pulmonary function waveforms and photovolumetric pulse waves | 62 | 360 | 360 | 10-fold cross-validation | 279 |
| S. D. Almeida | 2024 | Germany | Cohort study | Multicenter | Diagnosis | CT images | 7549 | 7549 | 4528 | 10-fold cross-validation | 1133 |
| E. Christina Dally | 2024 | India | Cohort study | Registry database | Classification | Clinical and pulmonary function data |  | 101 | 80 | Internal validation | 21 |
| M. F. A. Chaudhary | 2024 | United States | Cohort study | Multicenter | Diagnosis | CT images | SPIROMICS cohort: 1458; COPDGene cohort: 458 | SPIROMICS cohort: 2981; COPDGene cohort: 10305 | 1055 | External validation (Multicenter) | 458​ |
| N. Cai | 2024 | China | Case-control study | Single center | Diagnosis | CT images |  | 161 | 110 | 5-fold cross-validation | 51 |
| S. R. Alve | 2024 | Bangladesh | Case-control study | Single center | Classification and diagnosis | Breath sounds |  | 126 | 113 | 10-fold cross-validation | 13 |
| S. D. Almeida | 2024 | Germany | Cohort study | Multicenter | Diagnosis | CT images |  | 3144 | COPDGene training set: 3144 cases (including normal and disease groups) Generative model training set: 1373 cases (only "normal" people, GOLD0, and healthy individuals) | Internal validation External validation | COPDGene validation set: 786 cases COSYCONET test set: 446 cases |
| A. Roy | 2025 | India | Cohort study | Multicenter registry database | Classification and diagnosis | Audio features | 724 | 1120 |  | 5-fold cross-validation |  |
| S. H. Mei | 2025 | China | Cohort study | Registry database | Diagnosis | Pulmonary function curve | 19,308 | 348,039 | 278,431 | External validation | 69,608 |
| S. C. Feng | 2025 | China | Cohort study | Single center | Classification | CT images | 113 | 219 | 153 | Internal validation | External validation set: 29 cases; internal test set: 66 cases |
| T. Dorosti | 2025 | Germany | Case-control study | Single center | Diagnosis | Fleischner score | 99 | 23 | 7 | Internal validation (random sampling) | 12 |
| S. Feng | 2025 | China | Cohort study (retrospective) | Multicenter | Diagnosis | The pulmonary function indicators of the GOLD standard, including the FEV1/FVC ratio and the percentage of FEV1 | 223 | 2823 | 59 | Internal validation (random sampling), external validation(multicenter, retrospective) | External validation of diagnostic models: 50; external validation of classification models: 29 |
| A. K. M. S. A. Rabby | 2025 | United States | Cohort study (retrospective) | Multicenter | Diagnosis | FEV₁/FVC < 0.70 | 5640 | 13,043 | 3993 | Internal validation (COPDGene test set)+external validation (multicenter) | 3877 |
| S. Rezvanjou | 2025 | Canada | Cohort study (retrospective) | Multicenter | Diagnosis | FEV₁/FVC < 0.70 | 821 | 1499 | 353 | 5-fold cross-validation, Separate internal testing, external multicenter testing | The validation fold samples were divided alternately from 742 people in the training set. The number of cases in the separate internal test validation set (internal test): 309. Number of cases in the external validation set: 448. |
| A. R. W. Sait | 2025 | Saudi Arabia | Retrospective cross-sectional diagnostic study | Multicenter | Diagnosis | The clinical diagnostic labels (COPD vs health) given in each public lung sound dataset were taken as the gold standard | 1401 | 1988 | / | 5-fold cross-validation | 112 |
| P. Sahu | 2025 | India | Cohort study (retrospective) | Multicenter | Diagnosis | The existing disease labels in the Respiratory Sound Database were adopted | 126 | 126 | / | / | / |


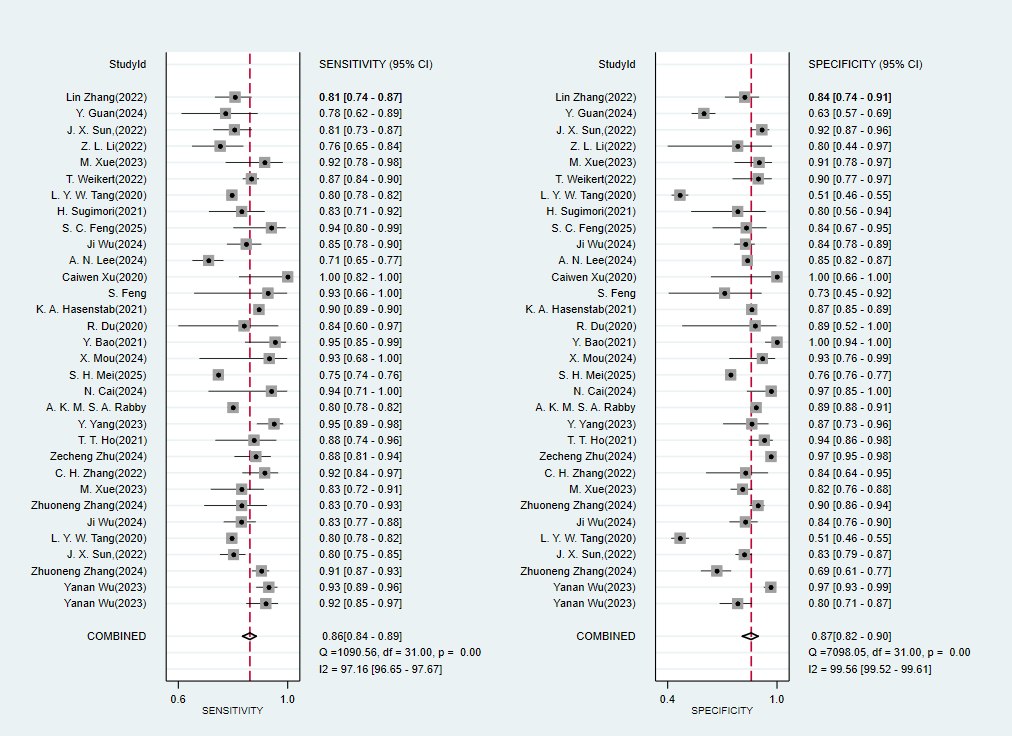


Figure S1 Forest plot of sensitivity and specificity of the CT-based DL model for binary classification of COPD, based on 30 validation cohorts from 14 countries between 2019 and 2025, relative to pulmonary function testing.


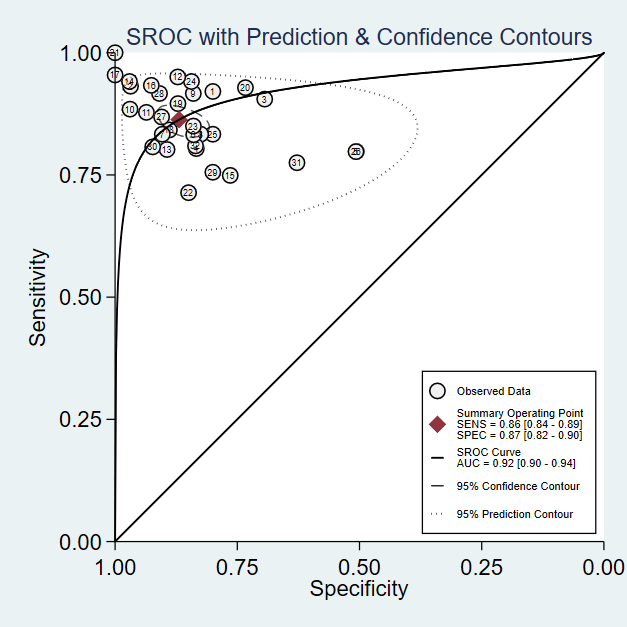


Figure S2 SROC curves of the CT-based DL model for binary classification of COPD, showing the overall diagnostic performance in each validation cohort from 14 countries between 2019 and 2025, relative to the gold standard of pulmonary function testing.


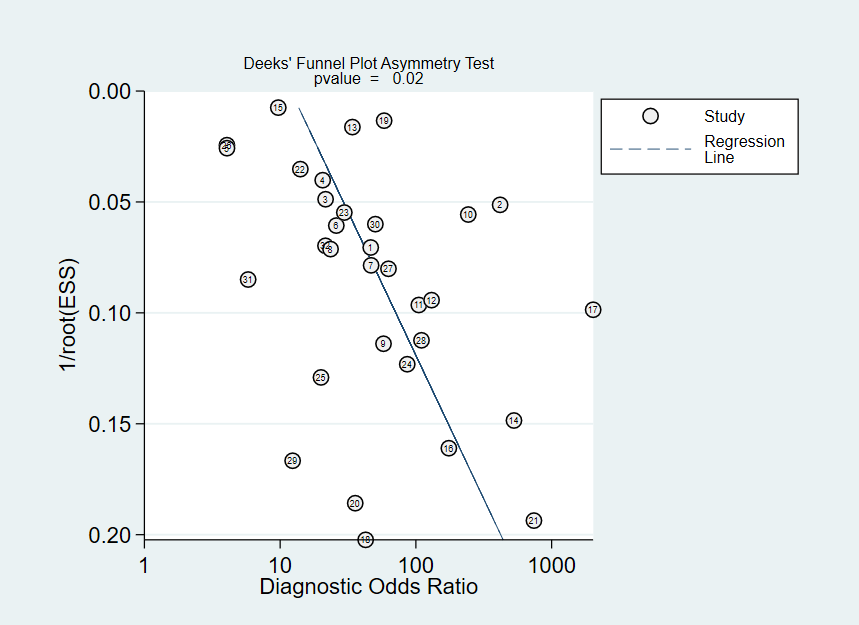


Figure S3 Deeks funnel plot of the CT-based DL model for binary classification of COPD, assessing publication bias and small-sample effects based on 32 validation cohorts.


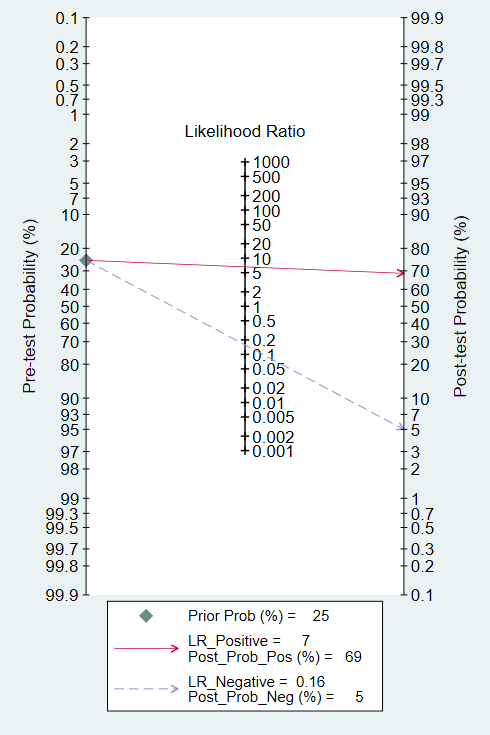


Figure S4 Fagan cursor plot of the CT-based DL model for binary classification of COPD, providing the posterior probabilities of positive and negative results based on the pooled likelihood ratio of 30 validation cohorts at a 25% pre-test probability.


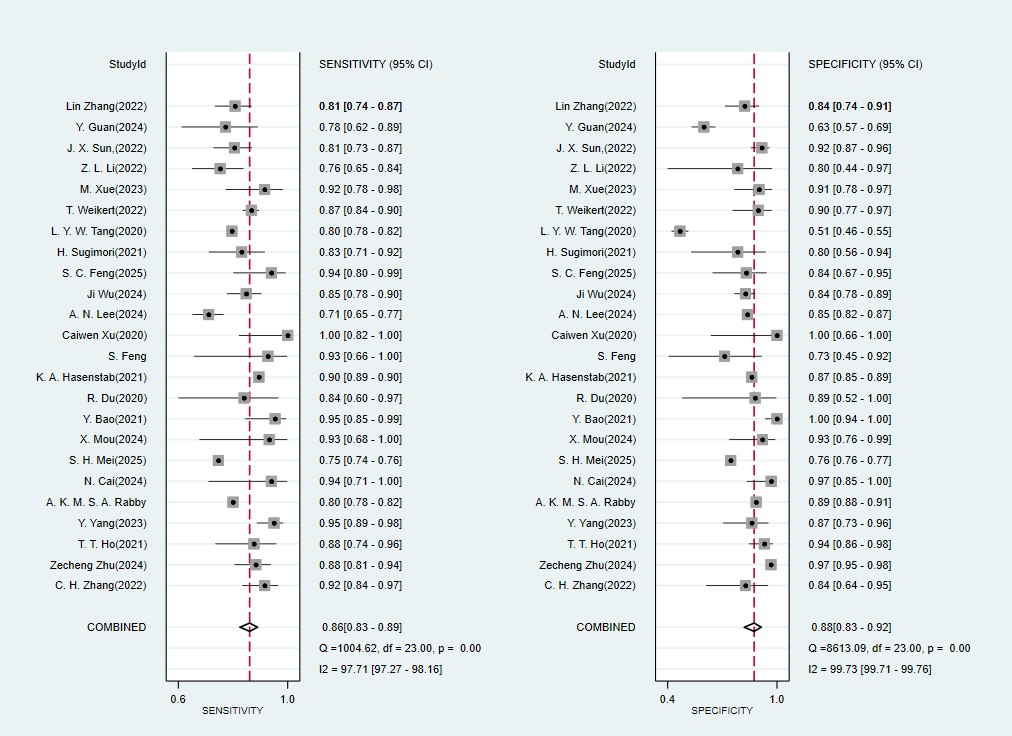


Figure S5 Forest plot of the CT-based DL model for binary classification of COPD in internal validation, summarizing the sensitivity and specificity of internal validation cohorts from 24 homologous datasets across 14 countries from 2019 to 2025.


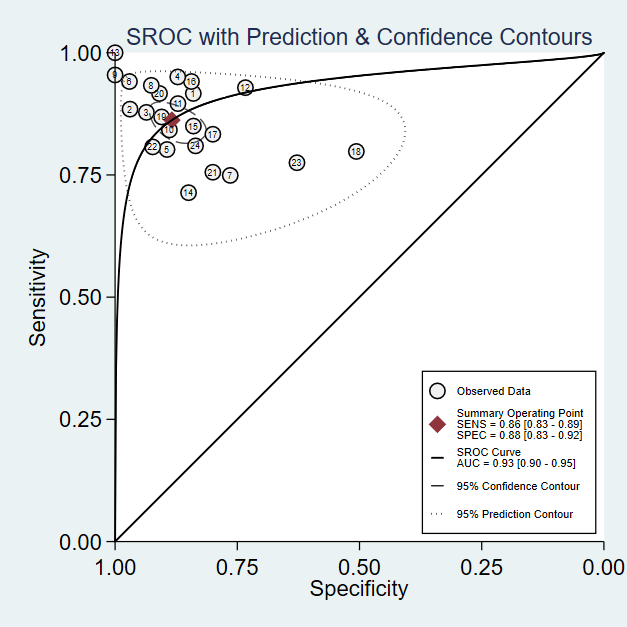
Figure S6 SROC curve of the CT-based DL model in internal validation, reflecting the overall diagnostic performance of the model across 24 internal validation cohorts, relative to pulmonary function testing.


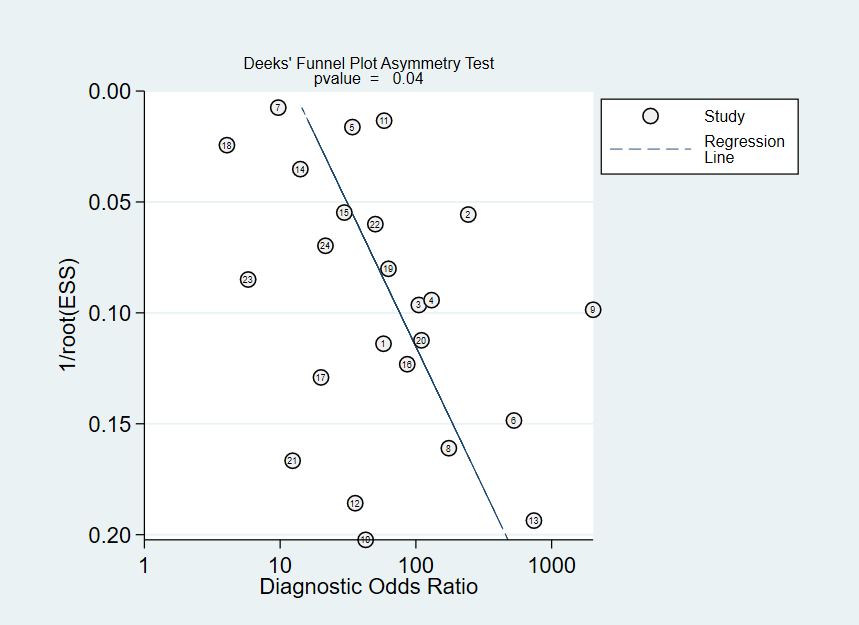
Figure S7 Deeks funnel plot for CT-based DL model in internal validation, assessing publication bias in the binary classification of COPD based on 24 cohorts; small sample effects may exist.


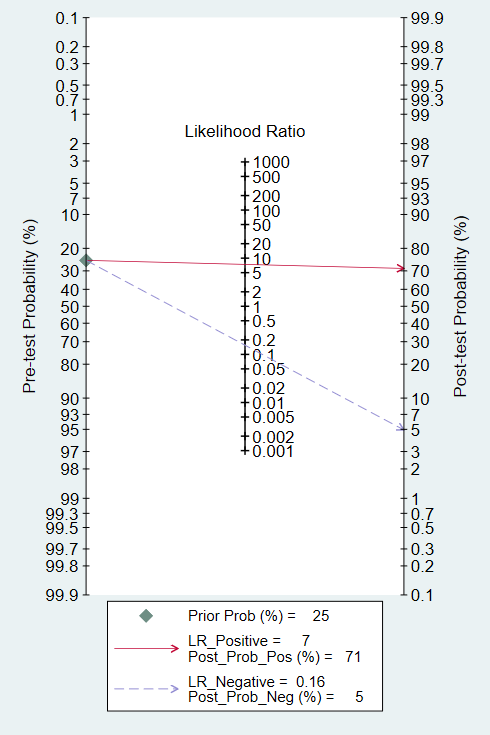


Figure S8 Fagan cursor plot for CT-based DL model in internal validation, estimating the posterior probability of COPD after positive/negative results using the pooled likelihood ratio of 24 internal validation cohorts at a 25% pre-test probability.


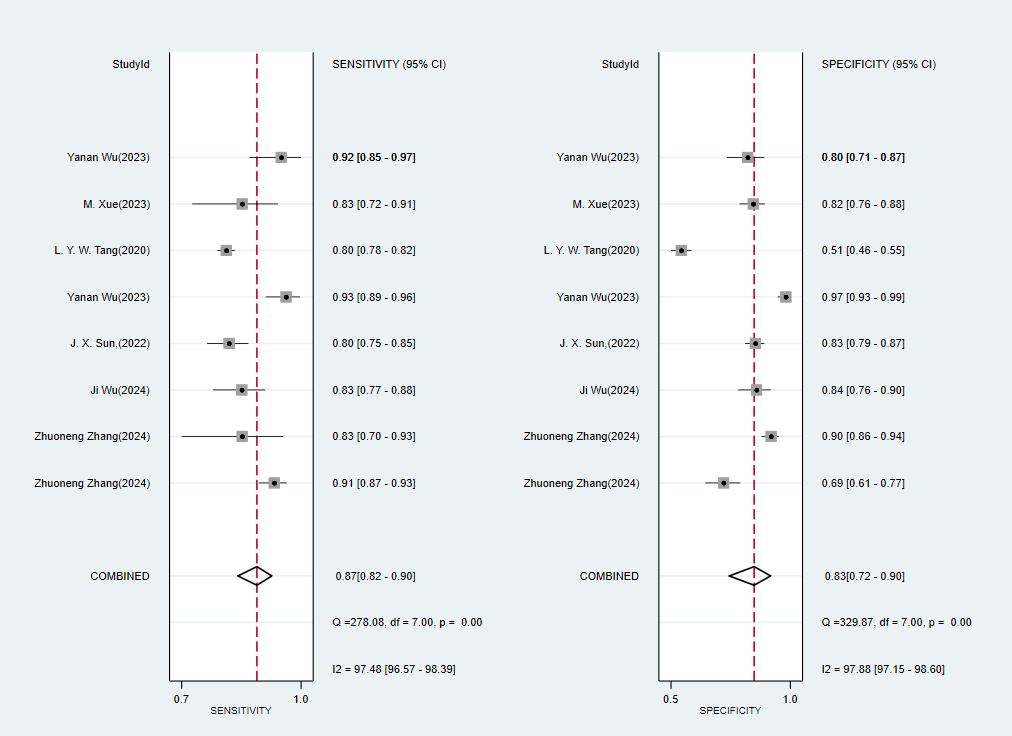


Figure S9 Forest plot for CT-based DL model for binary classification of COPD in external validation, summarizing sensitivity and specificity from 8 independent external datasets from 2019–2025.


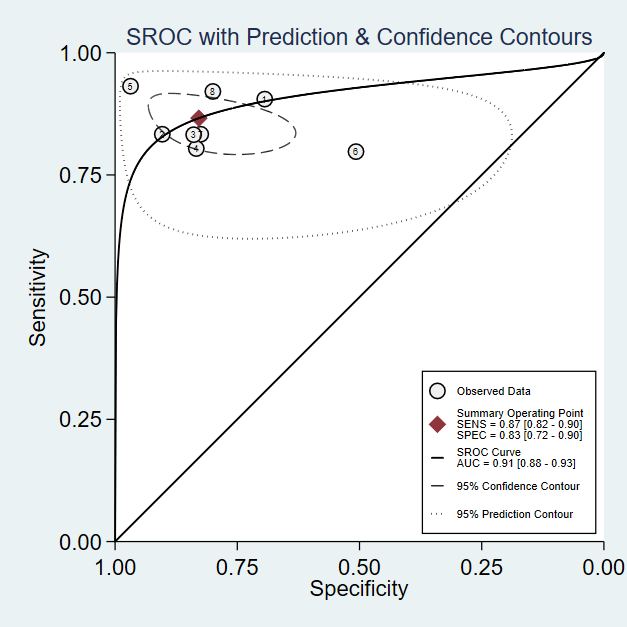


Figure S10 SROC curves of CT-based DL model in external validation, illustrating the overall diagnostic accuracy in the eight external validation cohorts, relative to the gold standard of pulmonary function testing.


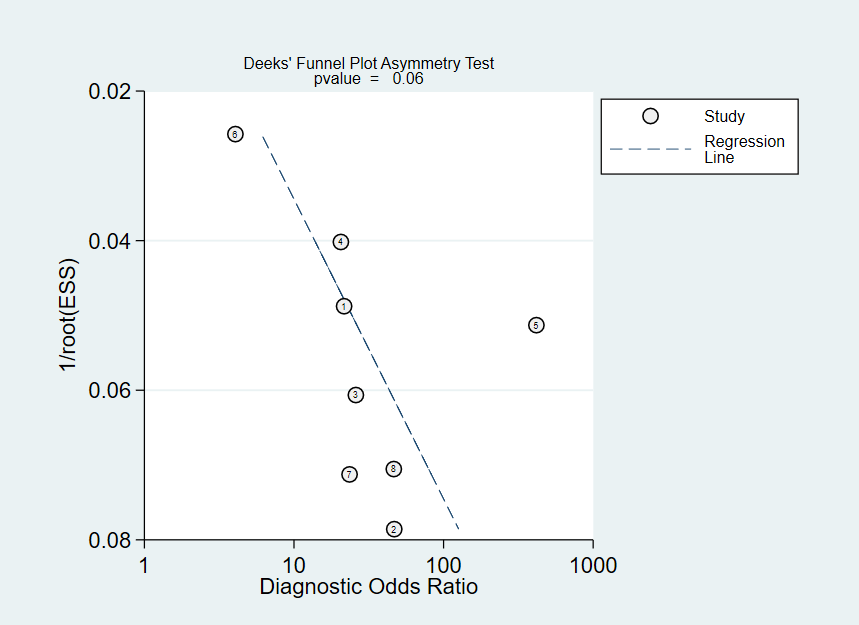


Figure S11 Deeks funnel plot of the CT-based DL model in external validation, assessing publication bias in the binary classification of COPD, based on eight cohorts.


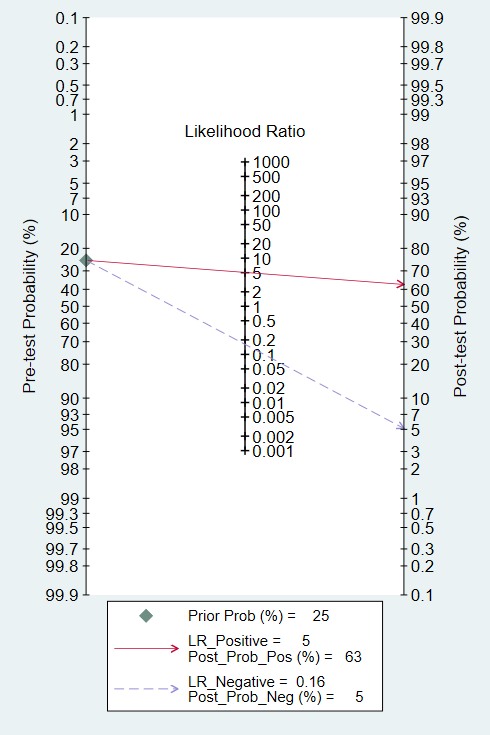


Figure S12 Fagan cursor plot of CT-based DL model in external validation, estimating the posterior probability of COPD after positive and negative results based on the pooled likelihood ratio of the eight external validation cohorts at a 25% pre-test probability.


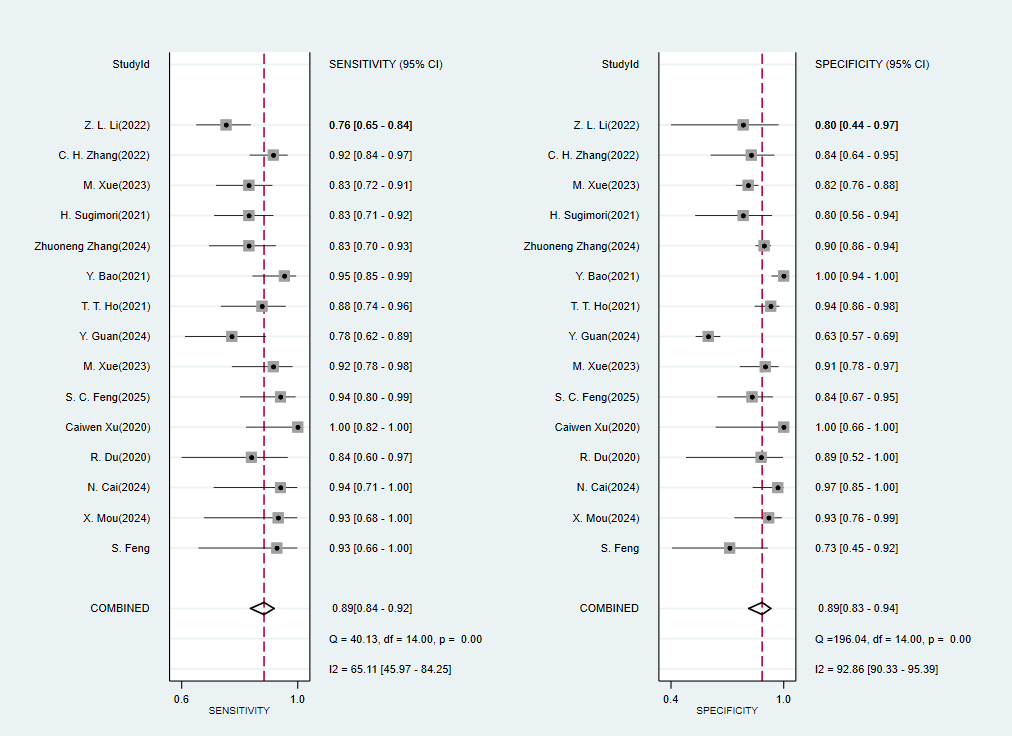


Figure S13 Forest plot of CT-based DL model for diagnosing COPD in the small-sample studies, summarizing the sensitivity and specificity of 15 validation cohorts to evaluate diagnostic performance in small-sample studies.


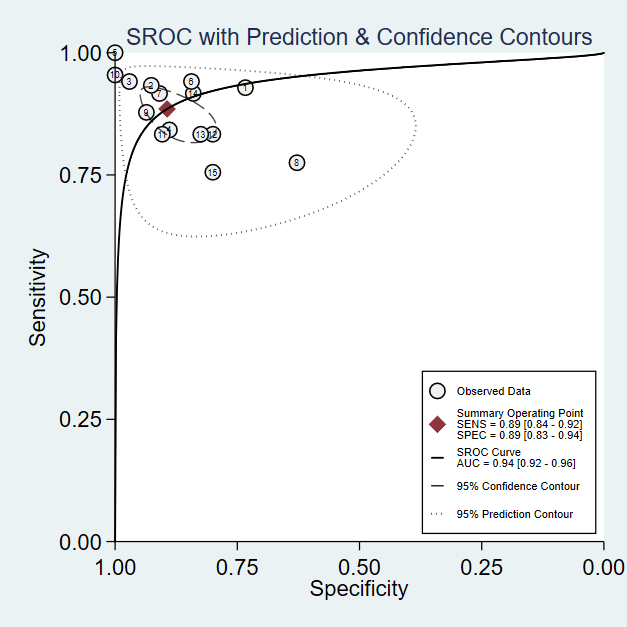


Figure S14 SROC curve of CT-based DL model for diagnosing COPD in the small-sample studies, based on 15 validation cohorts, summarizing the overall diagnostic efficacy (sensitivity-specificity tradeoff) in small-sample studies.
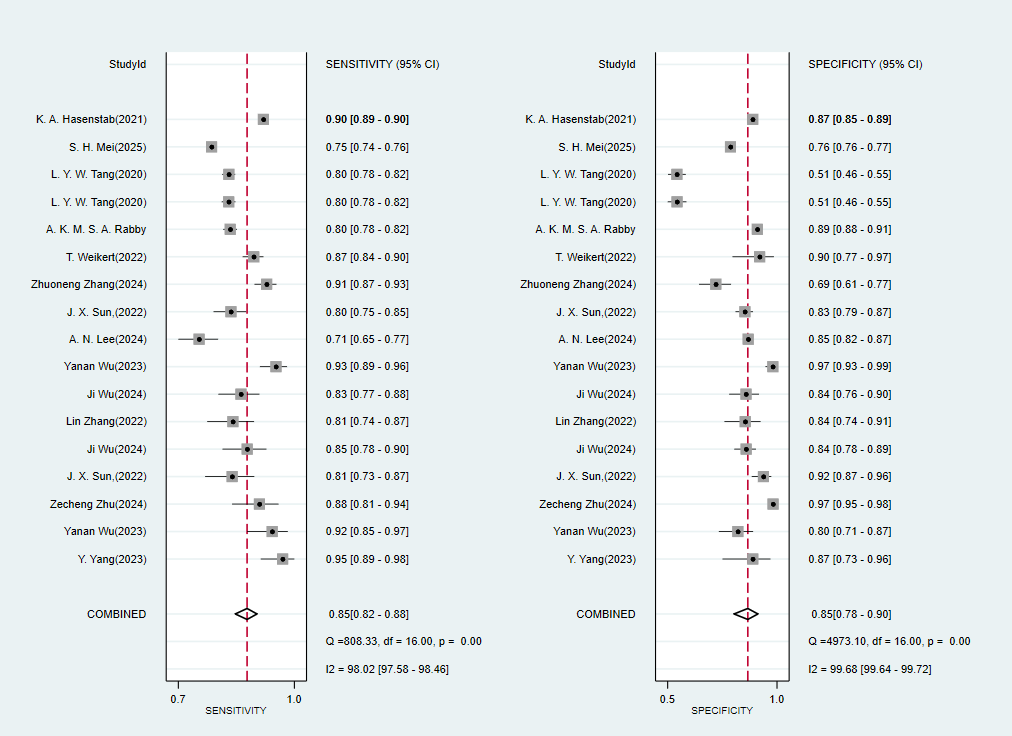


Figure S15 Forest plot for CT-based DL model for diagnosing COPD in large-sample studies. The sensitivity and specificity of 17 validation cohorts are summarized to evaluate diagnostic performance in large-sample studies.


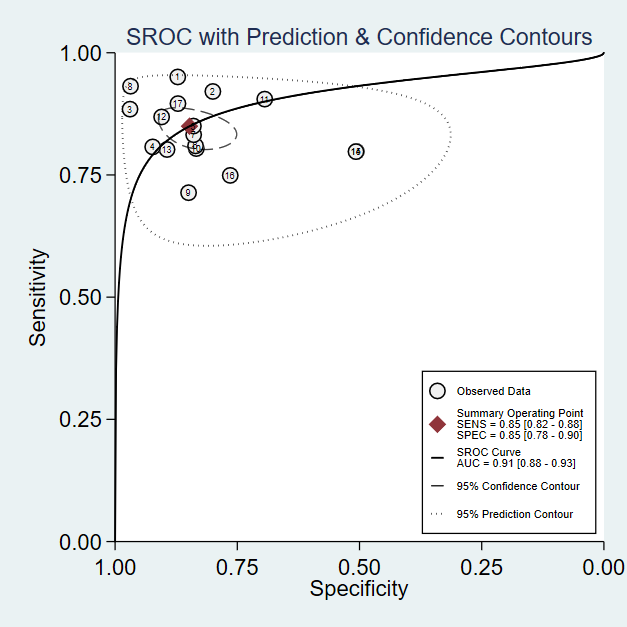


Figure S16 SROC curve for CT-based DL model for diagnosing COPD in large-sample studies, based on 17 validation cohorts, to evaluate diagnostic performance in large-sample studies.


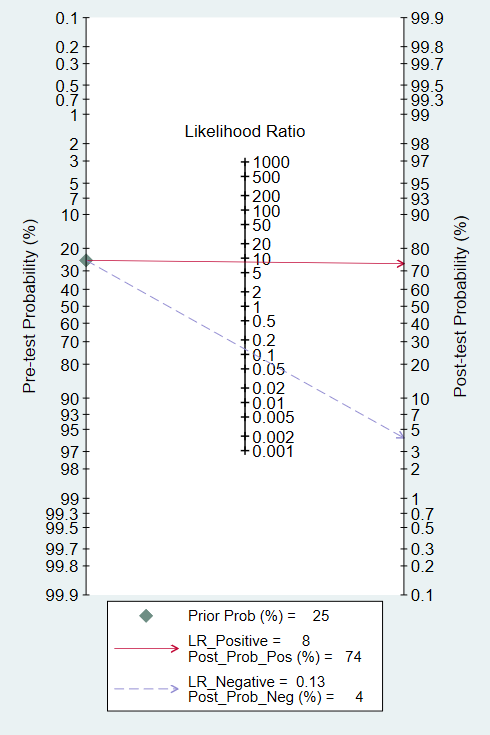


Figure S17 Fagan nomogram for CT-based DL model for diagnosing COPD in the small-sample studies. Assuming a given pre-detection probability, the pooled likelihood ratio of 15 validation cohorts shows the posterior disease probability corresponding to positive and negative results.


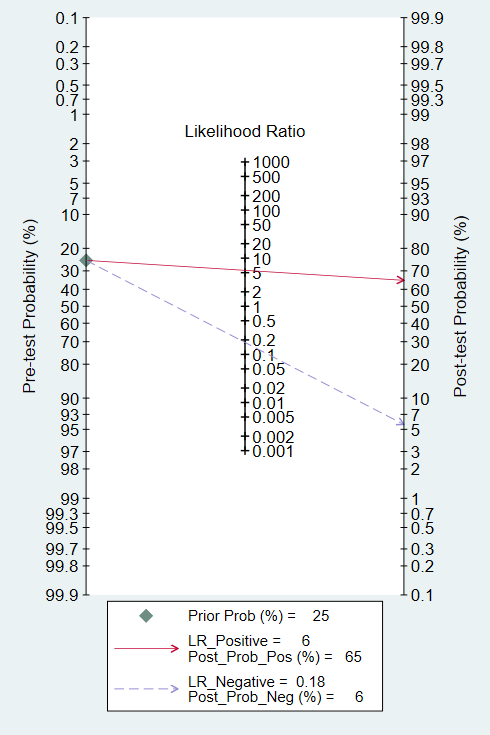


Figure S18 Fagan nomogram of CT-based DL model for diagnosing COPD in large-sample studies. The pooled likelihood ratio of 17 validation cohorts is used to show the posterior probability of positive and negative results under a pre-detection probability, to assess its clinical application potential.


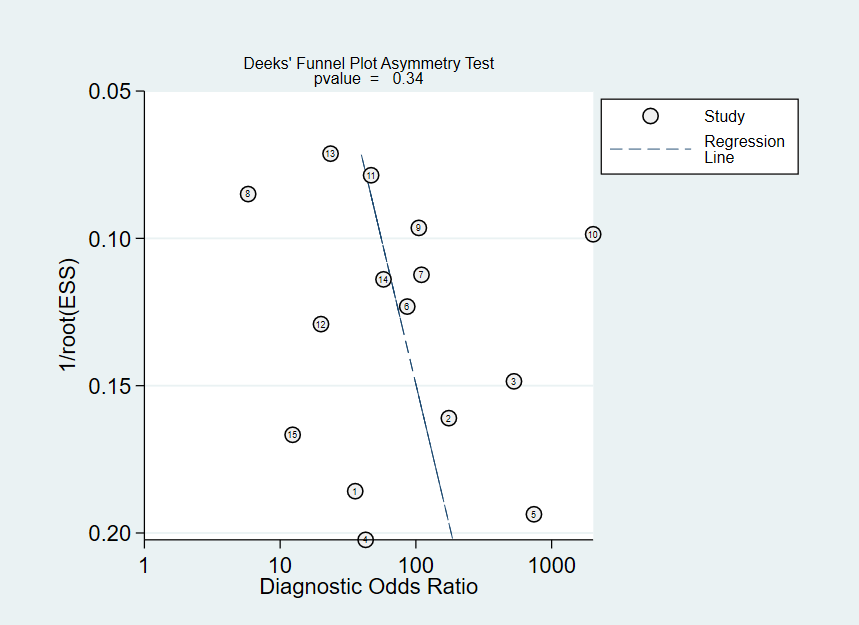


Figure S19 Deeks funnel plot of CT-based DL model for diagnosing COPD in the small-sample studies, based on 15 validation cohorts, used to examine for potential publication bias or small-sample effects in small-sample studies.


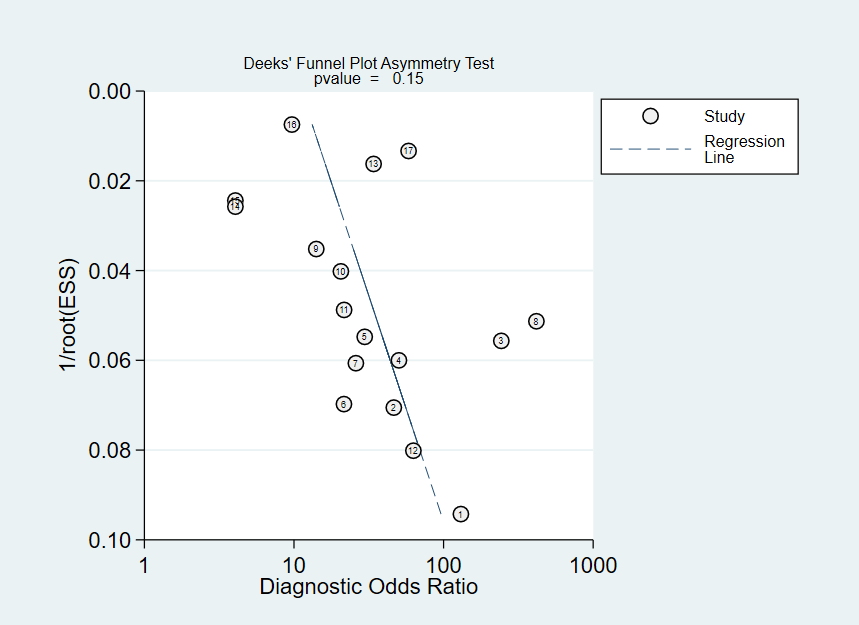
Figure S20 Deeks funnel plot of CT-based DL model for diagnosing COPD in large-sample studies, based on 17 validation cohorts, to assess publication bias in large-sample studies.


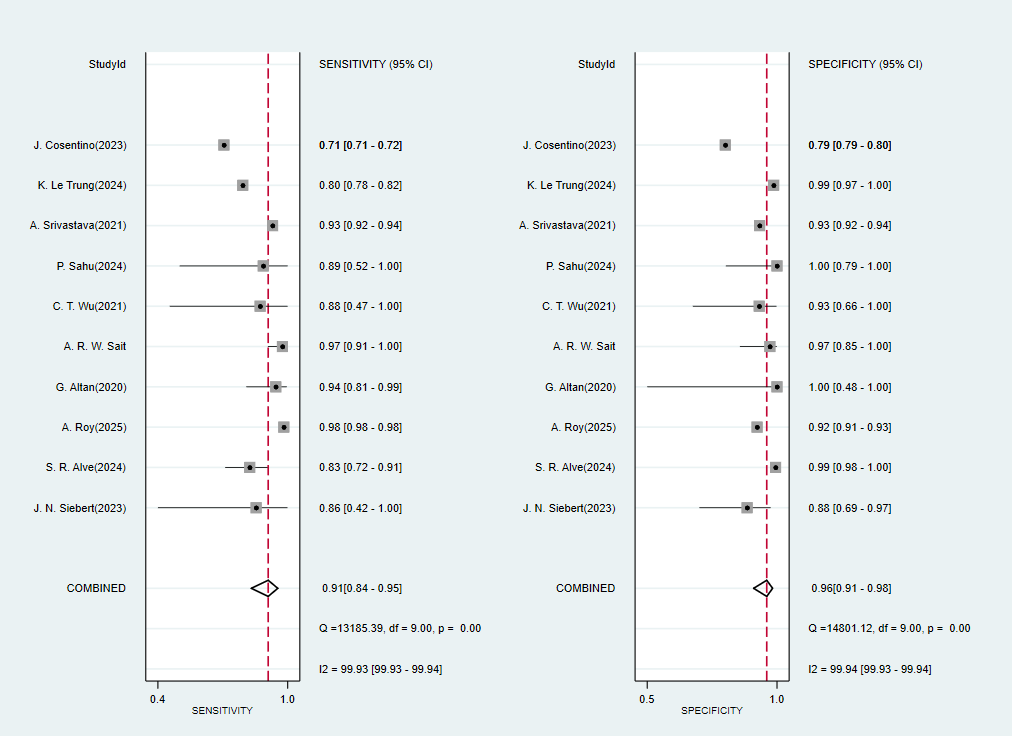


Figure S21 Forest plot of sensitivity and specificity of DL model based on breath sounds for diagnosing COPD, based on 10 validation cohorts from multiple countries from 2019–2025. Breath sounds were obtained from electronic stethoscopes or public databases, relative to pulmonary function testing.


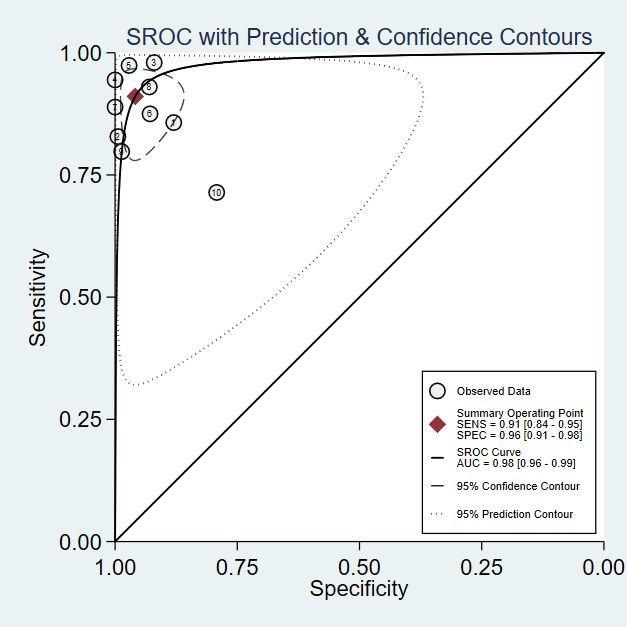


Figure S22 SROC curve for the DL model based on breath sounds in the binary classification of COPD, summarizing the overall diagnostic performance across 10 validation cohorts, relative to pulmonary function testing.


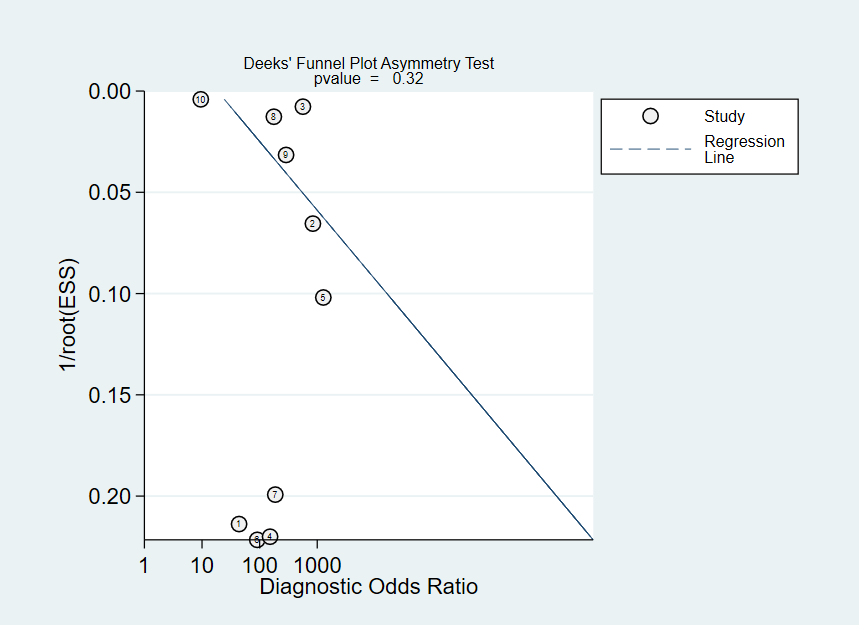
Figure S23 Deeks funnel plot for the DL model based on breath sounds in the binary classification of COPD, assessing publication bias based on 10 validation cohorts, with no significant small sample-effect observed.


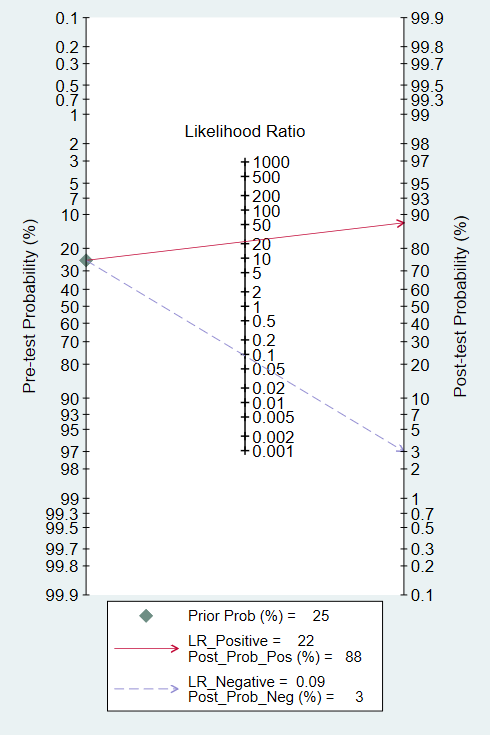


Figure S24 Fagan cursor plot for the DL model based on breath sounds in the binary classification of COPD, providing the posterior probability of positive/negative results based on the pooled likelihood ratio of 10 validation cohorts, under a 25% pre-test probability.


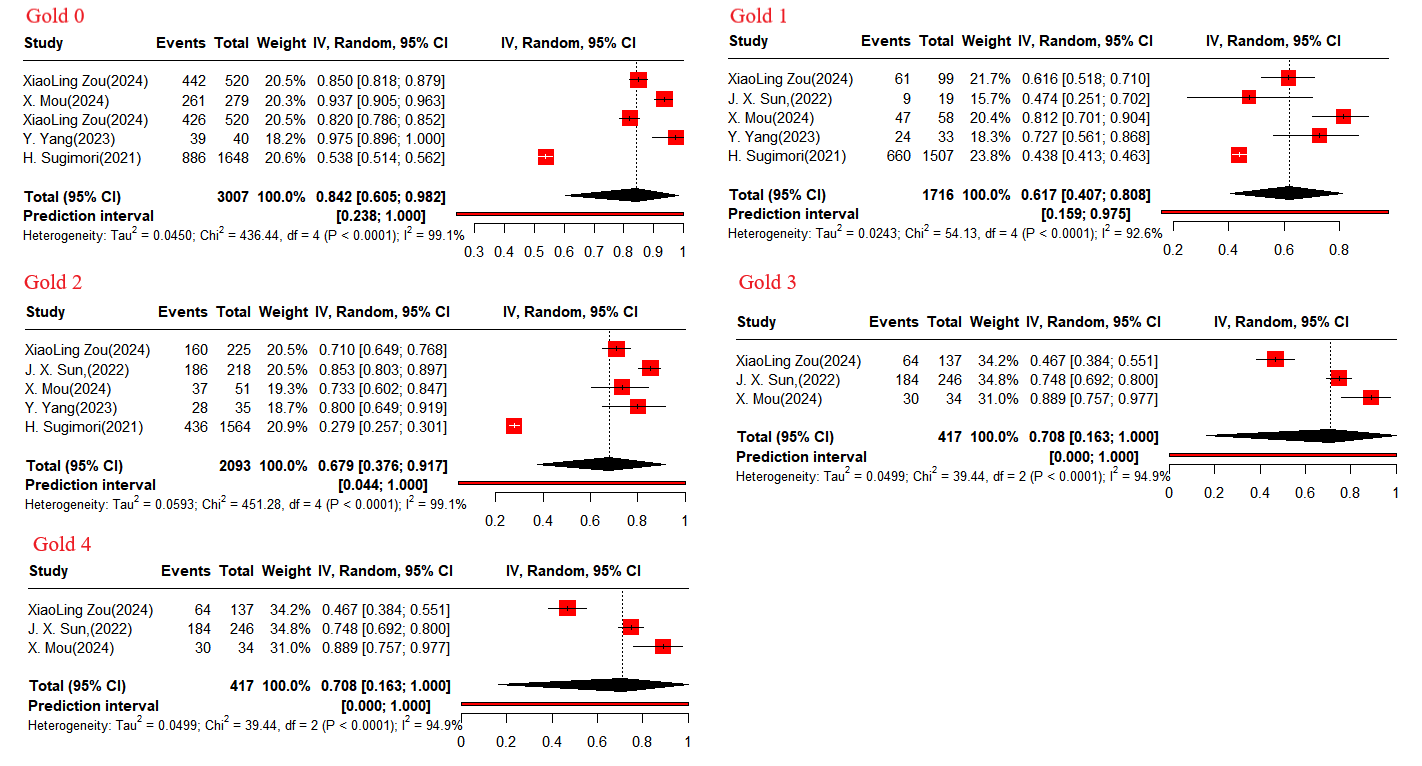


Figure S25 Forest plots of the accuracy of DL models for multiclass GOLD grading of COPD (GOLD 0–4), based on CT-derived validation cohorts, showing study-specific and pooled estimates with 95% confidence intervals and prediction intervals using a random-effects model with HKSJ adjustment.
